# Molecular Dynamics Study on Combustion Characteristics and Soot Formation Reactions of C_4_ Isomers Blended with NH_3_

**DOI:** 10.3390/molecules31213893

**Published:** 2026-10-28

**Authors:** Yang Wang, Jinhao Chen, Junjie Lu, Zhenhui Zhang, Yiju Zhao, Lumin Chen, Mingyan Gu

**Affiliations:** 1 Maanshan General Hospital of Ranger-Duree Healthcare, Ma’anshan 243000, China; 2 Engineering Research Center of Biofilm Water Purification and Utilization, Technology of Ministry of Education, Anhui University of Technology, Ma’anshan 243032, China; 3 School of Mechanical and Electrical Engineering, Tongling University, Tongling 244061, China

**Keywords:** C_4_ isomers, NH_3_-blended combustion, ReaxFF MD, soot formation

## Abstract

To achieve clean combustion of C_4_ hydrocarbon fuels, five typical C_4_ structural isomers were selected and reactive molecular-dynamics simulations with the ReaxFF force field were performed to compare combustion characteristics under neat-fuel and NH_3_-blended conditions. The results reveal strong structural dependence in C_4_ isomer combustion: terminal double bonds and tertiary C-H bonds govern ignition reactivity in the early stage. Carbon-skeleton cleavage controls overall reaction efficiency in the intermediate stage. Olefinic intermediates tend to persist in the late combustion phase, with distinct soot-precursor distributions across fuels. Ammonia addition markedly shortens oxygen-consumption duration for all systems, suppresses CO_2_ formation, and increases H_2_ and H_2_O yields. For soot formation, neat olefins produce far more six-membered rings than alkanes. NH_3_ blending raises the system-level C/H atomic ratio, and nitrogen atoms are incorporated into soot skeletons via nitrogen-containing functional groups.

## 1. Introduction

Nowadays, environmental challenges including global warming, frequent extreme meteorological events and aggravated atmospheric pollution have become critical bottlenecks restricting the advancement of human society [1,2,3]. Combustion, the core technology for energy conversion, dominates energy supply and represents the primary contributor to greenhouse gas and pollutant emissions [4,5]. Amid the transition of global energy mix, ammonia (NH_3_) is recognized as a highly promising alternative fuel due to its carbon-free property and mature storage and transportation technologies [6,7]. Additionally, NH_3_ possesses a volumetric energy density comparable to conventional fossil fuels along with a superior octane number, which enables its practical engineering deployment [8]. However, pure NH_3_ combustion is plagued by inherent limitations such as high ignition energy, low burning velocity and poor flame stability, which restricts its standalone application as fuel [9]. In addition, the combustion of pure NH_3_ tends to produce nitrogen-containing pollutants such as NO, NO_2_ and N_2_O. Under fuel-rich conditions, highly toxic HCN can also be formed, causing irreversible damage to the human body [10,11].

C_4_ hydrocarbons serve as major byproducts generated during catalytic cracking and steam cracking processes in the petroleum refining industry [12,13], and they also constitute the core components of liquefied petroleum gas. At present, C_4_ resources are primarily utilized as low-grade fuels with a low chemical conversion rate. This approach brings about a series of drawbacks including low added value of resources, inefficient combustion utilization, and great difficulties in the control of pollutant emissions [14,15]. By contrast, the co-combustion of C_4_ isomers with NH_3_ delivers dual benefits: high-value utilization of C_4_ resources and large-scale practical application of NH_3_ fuels [16]. Such blended fuels can be widely deployed in various engineering facilities such as industrial furnaces, internal combustion engines, and gas turbines, thereby possessing prominent prospects for engineering [5,17].

In recent years, investigations into the combustion of C_4_ isomers have covered multi-scale analyses ranging from macroscopic flame behaviors to microscopic radical reaction kinetics. Chen et al. [18] adopted an opposed-flow diffusion flame configuration and observed the transition of flames fueled by 1-butene, isobutene, n-butane and isobutane from a soot-free state to a heavily sooting state by gradually increasing the fuel mole fraction in the fuel stream. Experimental results indicated that the critical fuel concentrations required to reach the macroscopic soot transition threshold were 9.0% for 1-butene, 9.5% for isobutene, 19% for isobutane and 25% for n-butane. This concentration sequence demonstrates that C_4_ olefins exhibit a far greater tendency for soot formation than their corresponding alkanes. Moreover, the branching structure exerts a non-monotonic effect on the soot propensity of alkanes and olefins. For alkanes, branching facilitates soot generation, whereas linear olefin structures possess higher soot-forming tendencies than branched counterparts. Zhang et al. [19] explored the pyrolysis processes of three butene isomers at low pressure within a temperature range of 900~1900 K by employing synchrotron vacuum ultraviolet photoionization mass spectrometry. Reaction flux analysis clarified the dominant decomposition pathways of the three isomers. It was also revealed that the linear double-bond position and branched structure of C_4_ olefin isomers substantially alter their initial decomposition routes and product distribution profiles.

Co-combustion of hydrocarbon fuels blended with NH_3_ can remarkably improve the combustion performance and pollutant formation characteristics of pure NH_3_. Guo et al. [20] measured and compared the laminar burning velocities of NH_3_ mixtures doped with n-heptane, gasoline and RP-3 kerosene. The results demonstrated that the n-heptane/NH_3_ blended fuel possessed the highest laminar burning velocity. This phenomenon was attributed to the straight short-chain structure of n-heptane, which facilitates dehydrogenation reactions to generate small hydrocarbon radicals that accelerate combustion reactions. Gain et al. [21] conducted experimental investigations on fuel-rich jet flames fueled by C_2_H_6_/NH_3_ mixtures. Their observations revealed that increasing the NH_3_ blending ratio systematically reduced the spectral emission intensities of CH, OH and C_2_, which are characteristic hydrocarbon intermediates. In contrast, the emission intensity of CN, nitrogen-containing intermediates, increased significantly. Further mechanistic analysis indicated that NH_3_ addition suppresses the propagation of ethane oxidation chains while promoting the formation of HCN and CN species, thereby lowering the yield of soot precursors. Chen et al. [22] explored the gas–solid coupling mechanism during the co-pyrolysis of C_4_ isomers and NH_3_ in a tubular furnace at 1473 K, and uncovered the dual chemical effects of NH_3_ on soot inhibition. From the perspective of gas-phase chemistry, both experimental measurements and numerical simulations confirmed that NH_3_ addition reduces the concentrations of critical soot precursors including C_2_H_2_, C_2_H_4_, and C_4_H_2_ in pyrolysis exhaust gas. Meanwhile, the concentration of benzene rises because the reaction pathways for benzene to convert into large polycyclic aromatic hydrocarbons (PAHs) are blocked. This can be explained by the fact that nitrogen-containing species decomposed from NH_3_ readily react with C_2_-C_4_ light hydrocarbons to form nitrogen–hydrogen compounds, which consume carbon atoms available for soot particle growth. In summary, the combustion behavior of neat C_4_ isomers and C_4_ isomer/NH_3_ blends is governed not only by C_4_ molecular structures and NH_3_ blending ratios, but also by microscale chemical interactions between hydrocarbons and NH_3_. Consequently, the soot formation mechanism under such blended combustion conditions remains highly complex and insufficiently understood, which necessitates further in-depth research.

As the most widely investigated binary fuel system in the field of NH_3_ co-combustion, NH_3_-CH_4_ serves as a research benchmark for understanding the fuel interaction of NH_3_-CH_4_ mixtures. Rocha et al. [23] studied premixed laminar flames of NH_3_-CH_4_–air mixtures under various pressures, *φ* and NH_3_ blending ratios. They identified a premixed-diffusion dual-flame structure within the flame. Both elevated pressure and increased NH_3_ fraction in the fuel suppress NO formation. Meanwhile, they proposed a new calculation method for laminar flame speed accounting for flow inhomogeneity and flame stretch. Jin et al. [24] adopted the Chemkin/Premix code combined with the Okafor reaction mechanism to investigate the combustion characteristics and reaction kinetics of high-pressure premixed NH_3_-CH_4_–air laminar flames. Their results revealed that increasing the initial pressure reduces the laminar burning velocity while raising the adiabatic flame temperature and net heat release rate. The rise in pressure lowers the peak concentrations of H, NH_2_, NO, NO_2_ and HCN radicals and inhibits NO production. Pashchenko et al. [25] carried out CFD modelling of the combustion of hydrogen-rich synthetic fuels in swirling flames and validated the model against experimental data. They systematically analyzed the effects of fuel composition on combustion and emission characteristics of swirling flames, providing references for the application of hydrogen-rich fuels in combustion devices.

It is evident that existing studies on NH_3_-CH_4_ co-combustion lay a solid foundation for NH_3_-C_4_ combustion research. Nevertheless, CH_4_ features a simple molecular structure, and the effect of NH_3_ exhibits relatively limited diversity. In contrast, C_4_ fuels have a variety of isomeric structures, and NH_3_ exerts dual effects on the combustion of C_4_ fuels: it suppresses partial fuel pyrolysis via radical competition, while hydrogen production from NH_3_ decomposition accelerates the reactions of certain fuel components. Meanwhile, CH_4_ combustion hardly generates soot, so most relevant investigations focus on NOx emissions. In comparison, C_4_ fuels produce substantial soot. In view of the above, this work selects five typical C_4_ isomers, namely n-butane, isobutane, 1-butene, 2-butene and isobutene, as research objects. Reactive molecular dynamics simulations are adopted to thoroughly investigate the combustion characteristics and soot formation mechanisms of neat combustion and NH_3_-blended combustion at the molecular level, as well as to analyze the evolution of species and radicals during combustion. This research provides theoretical support for the precise optimization of combustion technologies, facilitating the development of combustion systems with low emissions and high efficiency. In view of the above, five typical C_4_ isomers, namely n-butane, isobutane, 1-butene, 2-butene and isobutene, are selected as the research objects in this work. Reactive molecular dynamics (ReaxFF MD) simulations are adopted to deeply investigate the combustion characteristics and soot formation mechanisms of neat C_4_ fuels and C_4_/NH_3_ blends at the molecular scale. Moreover, the evolution rules of species and radicals throughout combustion are elaborated. This study can provide theoretical support for the precise optimization of combustion technologies, facilitating the development of combustion systems with low pollutant emissions and high energy efficiency.

## 2. Results and Discussion

### 2.1. Macroscopic Combustion Characteristics of C_4_ Isomers

**Figure 1:**
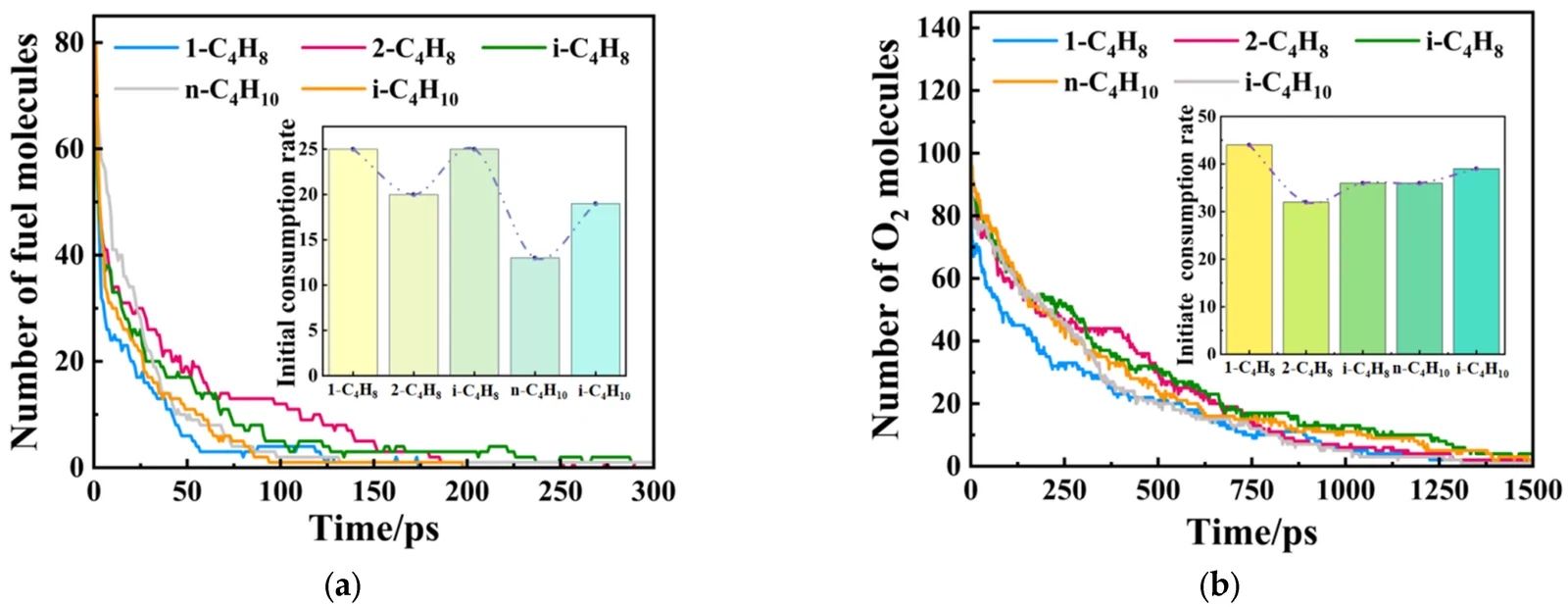
Evolution of residual molecular masses of C_4_ isomer fuels and O_2_ versus reaction time during combustion: (**a**) fuels, (**b**) O_2_.

The consumption rates of fuels and oxidants can reflect their combustion reactivity and macroscopic combustion behaviors. As illustrated in Figure 1a, all C_4_ isomers exhibit a consistent decreasing trend over time, while distinct discrepancies in consumption rates are observed at different reaction stages. The inset of Figure 1a displays the initial consumption rates of the tested isomers. It is evident that butene fuels possess remarkably higher initial consumption rates compared with butane counterparts. During the early reaction stage, 1-butene and isobutene are rapidly consumed via collisions with radicals including OH, H and O, benefiting from the low activation energy of double-bond addition and minor steric hindrance. In contrast, 2-butene suffers from severe steric hindrance induced by methyl groups on both sides of the carbon–carbon double bond. Despite the low energy barrier for addition reactions, its effective collision frequency is greatly suppressed. Among butane fuels, isobutane generates highly stable tert-butyl radicals through C-H bond cleavage with low activation energy for hydrogen abstraction, which yields a markedly faster initial reaction rate than n-butane.

After 50 ps, the dominant factor governing fuel consumption gradually shifts to the carbon skeleton cleavage and oxidation capacity of C_4_ isomers under high radical concentrations. At this stage, 1-butene still maintains the highest consumption rate. n-butane undergoes successive β-scission reactions via its linear carbon skeleton, generating abundant alkyl fragments that participate in chain propagation and subsequent oxidation processes. Its average consumption rate ranks second only to that of 1-butene. In comparison, isobutane reduces fuel concentration merely through rapid tert-butyl radical cleavage. Coupled with limited available decomposition pathways at high temperatures, its overall average consumption rate is lower than that of n-butane. As the reaction proceeds to approximately 100 ps, the discrepancy between butanes and butenes is further enlarged. At this moment, the consumption rates of butane fuels are remarkably lower than those of butene fuels. This is attributed to the absence of C=C double bonds in butane skeletons, which prevents the formation of resonance-stabilized structures and renders the carbon backbone more susceptible to oxidative breakdown. On the contrary, butenes generate intermediates with double bonds or allylic characteristics after undergoing addition, hydrogen abstraction and isomerization reactions. Such intermediates possess resonance stabilization effects and are resistant to cleavage under high-temperature environments with abundant radicals. Consequently, the residual molecular quantity of butene fuels remains higher than that of butanes.

Figure 1b depicts the evolution of residual O_2_ concentration over reaction time during combustion. The initial oxygen consumption rates, sorted in descending order, follow the sequence: 1-butene > isobutane > isobutene ≈ n-butane > 2-butene. Such disparities in reaction rates remain correlated with the molecular structures of fuels as elaborated above. 1-butene contains a terminal carbon–carbon double bond and highly reactive allylic H, which facilitates rapid generation of allylic radicals and their corresponding peroxy intermediates, thereby accelerating O_2_ consumption. Isobutane efficiently depletes O_2_ via small alkyl fragments yielded from tert-butyl radical cleavage. Although isobutene simultaneously possesses double bonds and branched carbon skeletons, substantial steric hindrance surrounding the C=C bond reduces the collision frequency between fuel molecules and O_2_. N-butane primarily consumes oxygen through hydrogen abstraction, β-scission, and peroxidation pathways. 2-butene exhibits the lowest double bond reactivity and steric accessibility among all tested C_4_ fuels. Moreover, the allylic radicals produced from 2-butene possess relatively high thermodynamic stability, which slows down its reaction kinetics with O_2_ and results in the minimum initial O_2_ consumption rate. In summary, the combustion performance of C_4_ isomers exhibits pronounced structure-dependent characteristics. In the early reaction stage, highly reactive molecular sites dominate ignition behavior and O_2_ consumption rates. At the intermediate stage, carbon skeleton configurations govern the reaction rates of β-scission pathways and determine the sustained oxidation capacity of the fuel mixture. In the later combustion stage, resonance-stabilized allylic intermediates regulate the oxidation reactivity of residual fuel species.

**Figure 2:**
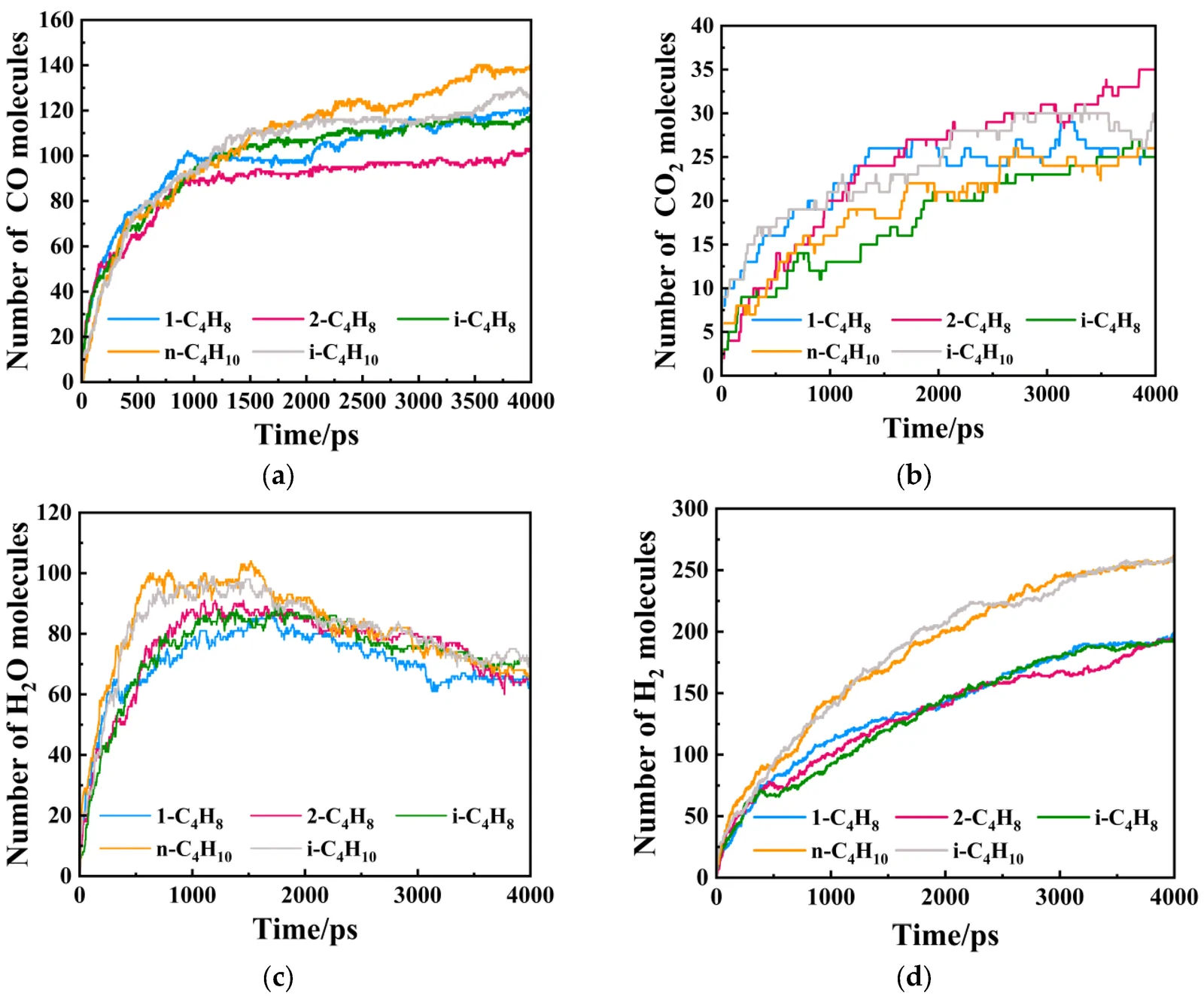
Temporal evolution curves of CO, CO_2_, H_2_O and H_2_ concentrations during neat combustion of C_4_ isomers: (**a**) CO, (**b**) CO_2_, (**c**) H_2_O, (**d**) H_2_.

C_4_ isomers exhibit dramatic discrepancies in molecular structure, branching degree and double-bond positions. Analysis of the dynamic evolution of combustion intermediates and final products can reveal fuel pyrolysis pathways, oxidation reaction rates and other core combustion characteristics [26,27]. Figure 2 displays the temporal evolution curves of CO, CO_2_, H_2_O, and H_2_ concentrations during the combustion of different C_4_ isomers. As illustrated in Figure 2a, butene fuels feature faster CO formation rates than butane fuels in the early reaction stage. After 750 ps, the CO generation rates of butane fuels rise sharply and gradually surpass those of butenes. Specifically, n-butane achieves the fastest growth in CO concentration: its CO yield first exceeds that of 2-butene, followed by isobutene and 1-butene in sequence. Isobutane presents a relatively gentle upward trend of CO concentration, yet its CO output outperforms all butene fuels in the late combustion stage. It is evident that butanes generate CO more readily as the reaction proceeds, despite their lower initial CO formation rates compared with butenes. Compared with CO, the generation profile of CO_2_ serves as a more reliable indicator of fuel oxidation capacity [27,28]. Figure 2b shows that isobutane delivers a rapid CO_2_ formation rate at the initial stage, which originates from the existence of tertiary C-H bonds in isobutane. The resulting tert-butyl radicals readily release methyl groups during subsequent oxidation and decomposition processes, which are further oxidized to form vital oxidation precursors. By contrast, 2-butene exhibits the slowest early-stage CO_2_ production. This indicates that its initial carbon conversion pathways fail to achieve complete oxidation, with most carbon skeletons retained in the form of incompletely oxidized intermediates, such as intermediate radicals and unsaturated pyrolysis fragments. As the reaction progresses, the CO_2_ generation rate of 2-butene rises continuously and eventually exceeds that of all other fuels. At the end of the simulation, the total CO_2_ yield follows the descending order: 2-butene > isobutane > n-butane ≈ 1-butene > isobutene. This ranking differs distinctly from the CO distribution. 2-butene yields the minimum amount of CO but the maximum amount of CO_2_, demonstrating its superior conversion efficiency toward fully oxidized products. In contrast, isobutene tends to form stable ketones and branched oxygenated intermediates. These species undergo complete oxidation at low kinetic rates, which accounts for its lowest final CO_2_ concentration among all tested C_4_ fuels.

The evolutionary characteristics of H_2_O presented in Figure 2c differ distinctly from those of CO and CO_2_. The molecular population of H_2_O exhibits an initial rising trend followed by a gradual decline, all simulated systems reach their H_2_O concentration peak at approximately 1250 ps, after which H_2_O concentration continuously decreases. This phenomenon indicates that H_2_O acts not only as a final combustion product but also as an intermediate species consumed in subsequent chemical reactions. From the perspective of molecular structure, each butane molecule contains two additional H compared with butene counterparts. Therefore, butanes convert H into H_2_O more readily during the early and intermediate reaction stages, yielding higher peak H_2_O concentrations. Subsequent consumption reactions then lead to a decline in H_2_O levels. Significant discrepancies in the net consumption of H_2_O are observed among different butene isomers, which are closely associated with reactive unsaturated oxygenated intermediates generated throughout the reaction. Such species continuously consume H_2_O formed in the combustion process.

The evolutionary trend of H_2_ differs markedly from that of H_2_O. As illustrated in Figure 2d, the concentration of H_2_ continuously rises over time during the combustion of all five C_4_ isomers without obvious decline, which indicates that H_2_ behaves much like a stable accumulated product. At the termination of the simulation, the total yield of H_2_ follows the descending order: n-butane > isobutane > 1-butene > 2-butene > isobutene. The higher H_2_ production from butane combustion originates from their greater intrinsic hydrogen storage capacity. Continuous dehydrogenation during butane pyrolysis persistently releases H, which facilitates the accumulation of H_2_. Among butene isomers, 1-butene generates more H_2_ than 2-butene and isobutene, demonstrating that the decomposition pathways of 1-butene are more favorable for H release and subsequent H_2_ formation. In contrast, 2-butene preferentially converts H into oxidized products such as H_2_O, resulting in a lower degree of H_2_ accumulation. Isobutene readily forms stable branched intermediates, a large quantity of H is trapped within organic intermediates with prolonged residence time, ultimately yielding the minimum H_2_ concentration among all tested fuels.

By comprehensively analyzing the evolutionary characteristics of four oxygen-containing and hydrogen-containing final products, namely CO, CO_2_, H_2_O and H_2_, it can be concluded that the combustion progress of C_4_ isomers is not merely governed by the ignition rate of fuels, but is synergistically regulated by multiple elemental reaction pathways. As a whole, butenes exhibit rapid initial reactions accompanied by prominent partial oxidation in the later stage, whereas butanes display a slightly slower initial reaction rate with more sustained oxidation throughout the subsequent combustion stage.

### 2.2. Effects of NH_3_ Addition on the Reactivity of C_4_ Isomers

**Figure 3:**
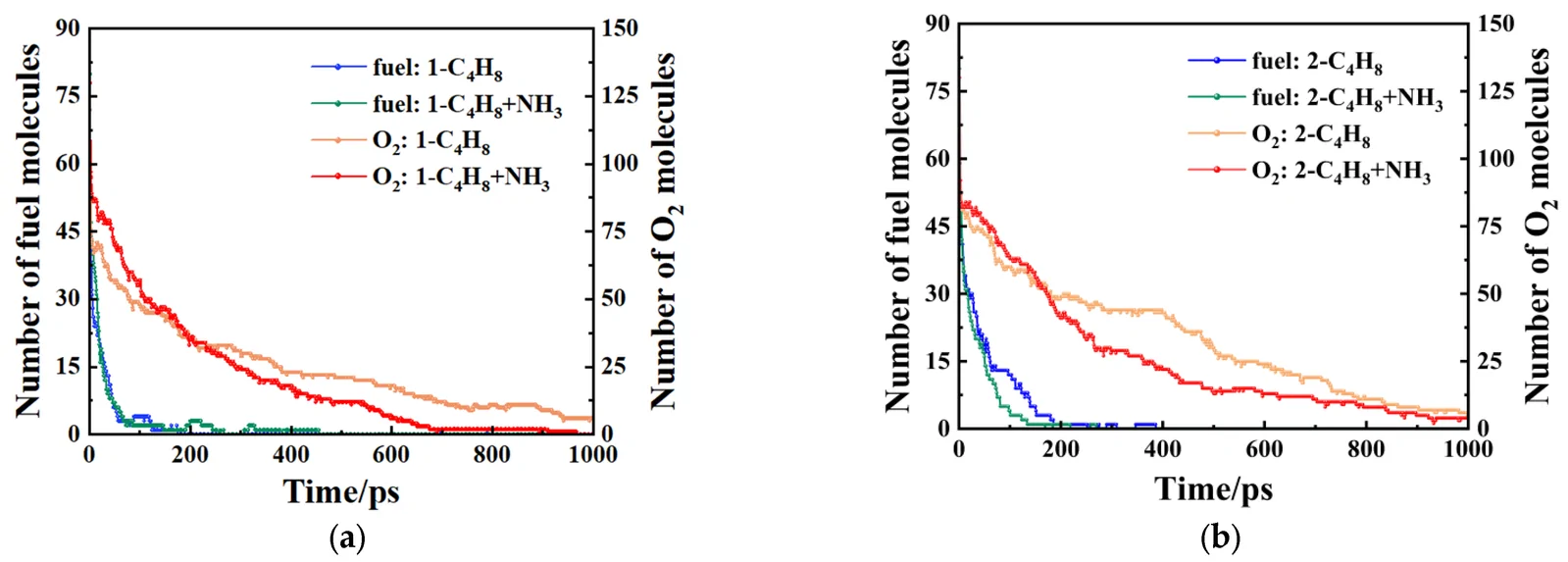

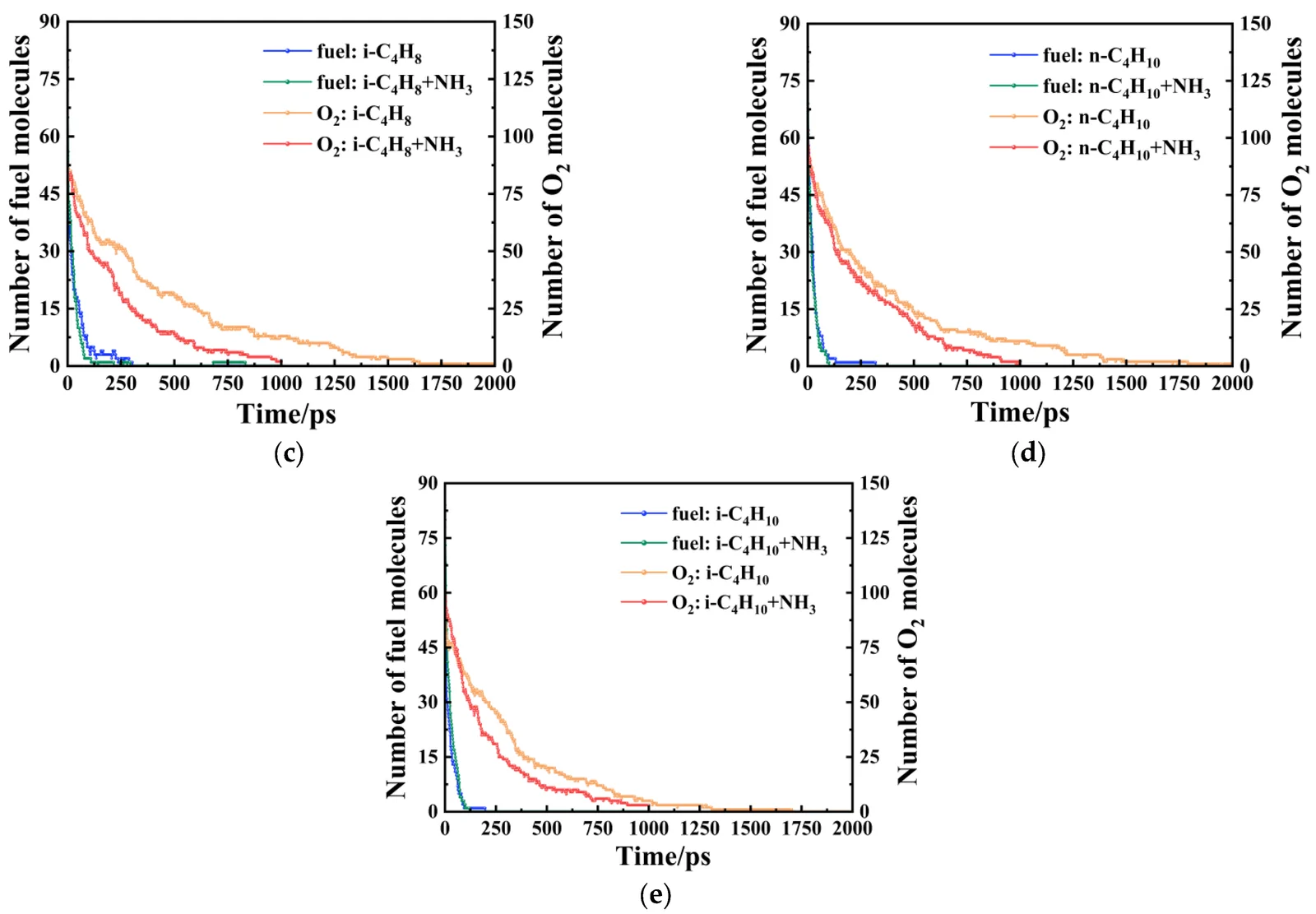
Temporal evolution of fuel and O_2_ concentrations during neat and NH_3_-blended combustion of C_4_ isomers: (**a**) 1-butene, (**b**) 2-butene, (**c**) isobutene, (**d**) n-butane, (**e**) isobutane.

Figure 3 illustrates the temporal evolution of fuel and O_2_ concentrations during neat combustion and NH_3_-blended combustion of C_4_ isomers. In this study, the blending ratio of NH_3_ and C_4_ molecules is fixed at 1:2, and detailed operating conditions are referred to Part 3. As presented in Figure 3a, neat 1-butene is consumed more rapidly within the initial 20 ps of the reaction. After 20 ps, the residual fuel amounts of the neat and NH_3_-blended cases gradually converge, and the fuels in both cases are completely depleted at approximately 130 ps. The discrepancy observed in the early reaction stage can be attributed to the competitive consumption of active radicals such as H, O and OH between NH_3_ and 1-butene. This competition slows chain reactions including double-bond addition, hydrogen abstraction and subsequent β-scission of the terminal alkene [29]. Nevertheless, this competitive effect only lasts for a short duration, once the radical pool becomes sufficiently abundant, the inhibitory impact of NH_3_ on 1-butene is substantially weakened. The molecular population of O_2_ for NH_3_-blended 1-butene combustion is higher than that of the neat case at the early reaction stage, indicating that NH_3_ also competes for oxidative radicals including O and OH. As the NH_3_ chain reactions proceed, O_2_ consumption is significantly accelerated. Ultimately, O_2_ is nearly exhausted at around 700 ps under the NH_3_-blended condition, much earlier than 1200 ps for neat combustion. In summary, although NH_3_ addition suppresses the initial pyrolysis of 1-butene, it introduces extra oxygen consumption pathways and elevates the overall O_2_ consumption rate.

Unlike 1-butene, NH_3_ blending remarkably accelerates the consumption of 2-butene. As shown in Figure 3b, 2-butene is completely depleted at approximately 180 ps under the NH_3_-blended condition, while full fuel consumption is not achieved until 250 ps for neat combustion, demonstrating that NH_3_ exerts a promoting effect on the pyrolysis of 2-butene. On one hand, NH_3_ competes for partial radicals as elaborated in the preceding section. On the other hand, 2-butene is categorized as an internal alkene with its C=C double bond located at the middle of the carbon chain, which leads to severe steric hindrance and relatively sluggish initial reactivity [30,31]. Atomic hydrogen generated from the decomposition of blended NH_3_ participates in reactions at an early stage and compensates for the insufficient initial reactivity of internal alkenes. Since the reactivity-enhancing effect derived from the structural characteristics of 2-butene outweighs the radical competitive effect, the overall consumption rate of 2-butene is elevated after NH_3_ addition. The initial molecular population of O_2_ in the NH_3_-blended 2-butene system is higher than that of the neat case, which indicates that NH_3_ competes for oxidative radicals in the early reaction stage. As the reaction proceeds, however, the O_2_ consumption rate of the blended system rises drastically. The O_2_ consumption rate surges after roughly 180 ps, and O_2_ is ultimately exhausted at nearly the same time as the neat combustion system. Accordingly, the influence of NH_3_ on O_2_ consumption during 2-butene combustion presents obvious stage-dependent characteristics.

The effects of NH_3_ blending on isobutene also exhibit obvious stage-dependent characteristics. As illustrated in Figure 3c, the fuel concentration of the neat case decreases more rapidly within the initial 30 ps of the reaction, which proves that NH_3_ still competes with isobutene for active radicals such as H, O and OH in the early stage. After 30 ps, the fuel consumption rate of the NH_3_-blended system gradually surpasses that of the neat system, revealing that radicals supplemented by decomposed NH_3_ can promote chain reactions. Eventually, the fuel in both cases is completely depleted at approximately 300 ps, indicating that NH_3_ blending barely alters the total fuel consumption duration of isobutene. The O_2_ consumption behavior of isobutene is more sensitive to NH_3_ addition. The O_2_ consumption rate is significantly accelerated after NH_3_ blending, with O_2_ nearly exhausted at around 1000 ps, whereas O_2_ is not fully consumed until roughly 1600 ps under neat combustion conditions. This phenomenon demonstrates that the oxidation chain reactions of NH_3_ are initiated rapidly in the isobutene system. O_2_ acts as the oxidant for isobutene and simultaneously participates in the oxidation of NH_3_. Accordingly, two parallel O_2_ consumption pathways—hydrocarbon oxidation and NH_3_ oxidation—function synergistically, accelerating the overall O_2_ consumption rate of the blended system.

As shown in Figure 3d, the fuel concentration curves of neat n-butane combustion and NH_3_-blended combustion are highly similar. The fuel is fully depleted at approximately 100 ps for the NH_3_-blended case, while complete consumption occurs at 130 ps under neat conditions, which indicates that NH_3_ only slightly promotes the pyrolysis of n-butane. NH_3_ blending imposes two counteracting effects on n-butane pyrolysis. On one hand, NH_3_ competes for active radicals including H, O, and OH, thereby suppressing the decomposition of n-butane. On the other hand, the decomposition of NH_3_ supplies atomic hydrogen, which accelerates hydrogen abstraction from C-H bonds and facilitates the progression of chain reactions. These two effects maintain a dynamic equilibrium, resulting in a marginal advance in the total burnout time. After NH_3_ addition, the O_2_ consumption rate of the n-butane system rises dramatically. O_2_ is nearly exhausted at roughly 1000 ps, 1500 ps earlier than the neat combustion condition. Consequently, NH_3_ blending exerts a negligible influence on n-butane consumption yet imposes a remarkable accelerating effect on O_2_ depletion.

NH_3_ blending suppresses the early-stage consumption of isobutane. As illustrated in Figure 3e, the molecular population of isobutane under the NH_3_-blended condition is initially higher than that of neat combustion before both curves converge, which is correlated with the molecular structure of isobutane. Isobutane contains tertiary C-H bonds with low bond dissociation energies, enabling rapid C-H bond cleavage and abundant radical accumulation at the initial stage of neat combustion. After NH_3_ addition, NH_3_ competes for active radicals in the early reaction stage, hindering isobutane pyrolysis and slowing down fuel consumption. As the reaction proceeds, continuous dehydrogenation of NH_3_ replenishes atomic hydrogen and intensifies chain reactions. Ultimately, full fuel consumption is achieved at approximately 100 ps for both working conditions. Thus, NH_3_ blending merely inhibits pyrolysis in the early stage and does not prolong the total fuel burnout duration. The variation in O_2_ consumption curves demonstrates that NH_3_ blending accelerates the O_2_ consumption rate in isobutane combustion. On one hand, the intrinsic oxidation reactions of NH_3_ create an additional O_2_ consumption pathway. On the other hand, nitrogen-containing radicals generated during the reaction participate in various elementary reactions and further expedite O_2_ depletion. In summary, NH_3_ blending restrains the early pyrolytic consumption of isobutane fuel but exerts a promoting effect on O_2_ consumption.

Overall, the effects of NH_3_ blending on C_4_ isomers exhibit pronounced molecular structure sensitivity [32]. For 1-butene and isobutane, the radical competitive effect of NH_3_ dominates the early reaction stage and suppresses the initial fuel consumption. In the case of 2-butene, atomic hydrogen generated by NH_3_ decomposition effectively accelerates hydrogen abstraction and pyrolysis of internal alkenes, thereby markedly speeding up fuel depletion. For isobutene and n-butane, NH_3_ blending primarily alters the stagewise consumption rate while exerting limited influence on the total time required for complete fuel consumption. O_2_ consumption shows higher sensitivity to NH_3_ addition. NH_3_ blending substantially shortens the total oxygen burnout time, which can be attributed to two factors. First, the oxidation reactions of NH_3_ create an extra O_2_ consumption pathway. Second, nitrogen-containing radicals generated during combustion participate in diverse elementary reactions and further expedite O_2_ depletion.

### 2.3. Effects of NH_3_ Addition on Combustion Products and Intermediates of C_4_ Isomers

**Figure 4:**
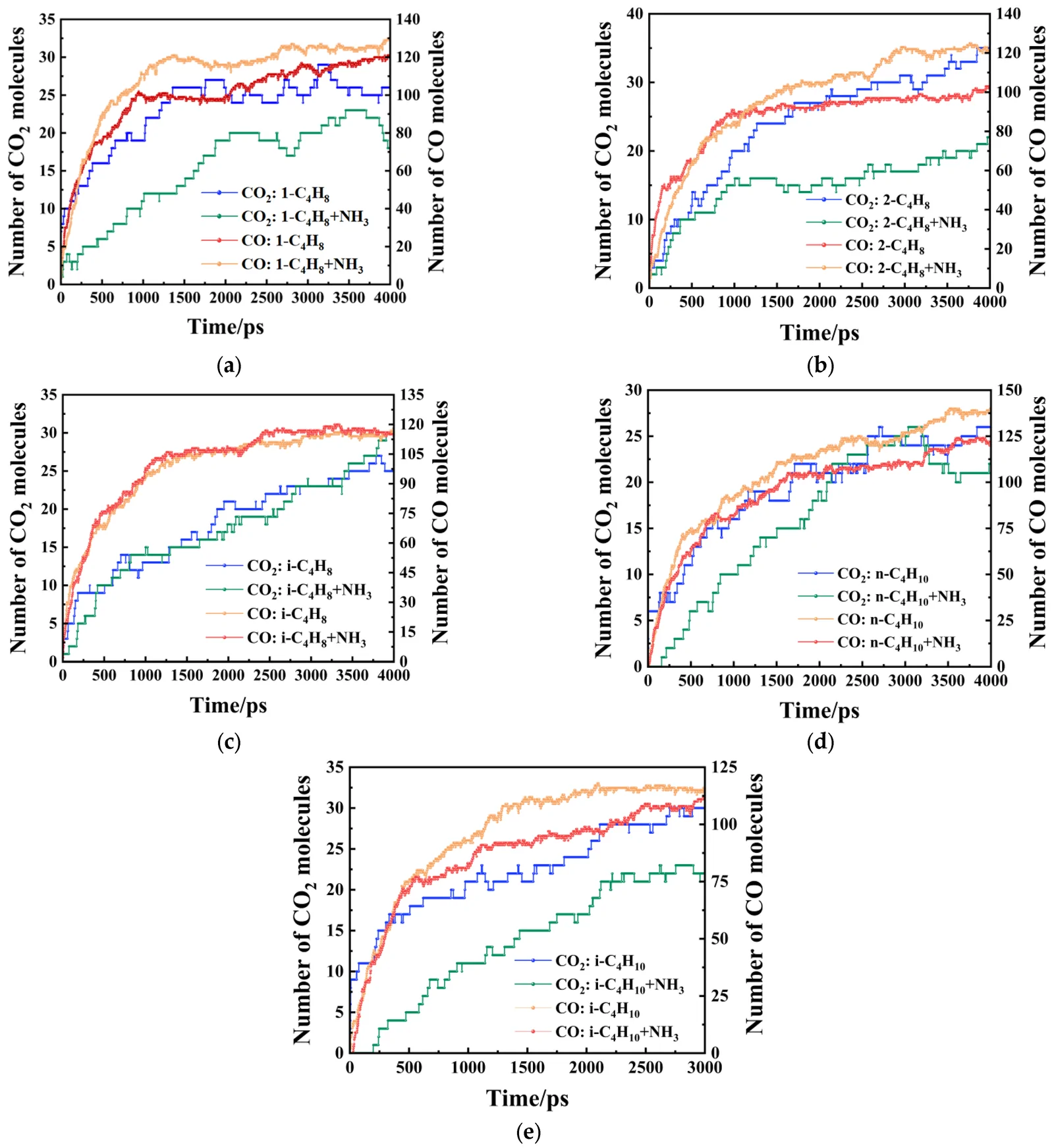
Temporal evolution of CO and CO_2_ during NH_3_-blended combustion of C_4_ isomers: (**a**) 1-butene, (**b**) 2-butene, (**c**) isobutene, (**d**) n-butane, (**e**) isobutane.

The formation rules of CO and CO_2_ reflect the carbon oxidation degree of C_4_ isomers. As illustrated in Figure 4a, within the initial 250 ps of NH_3_-doped 1-butene combustion, the CO formation rate is lower than that under pure combustion conditions. However, the number of CO molecules exceeds that of the pure combustion case after 250 ps. Meanwhile, the total yield of CO_2_ remains consistently lower throughout the entire reaction process when NH_3_ is blended. These results demonstrate that NH_3_ inhibits the initial oxidation of 1-butene, whereas it suppresses the conversion of CO to CO_2_ in the middle and later reaction stages, thereby lowering the overall carbon oxidation degree of the fuel.

As illustrated in Figure 4b, 2-butene exhibits analogous oxidation behaviors to 1-butene. For the NH_3_-doped combustion system, the mole number of CO remains consistently lower than that of the neat combustion case before 1200 ps, yet CO concentrations rise rapidly and surpass the neat counterpart after 1200 ps. Throughout the entire simulation duration, CO_2_ yields in the NH_3_-blended system are inferior to those of neat 2-butene combustion, with the concentration gap continuously widening after 1000 ps. Compared with 1-butene, the turning point where CO overtakes the neat baseline shifts distinctly to a later simulation time for 2-butene. This phenomenon demonstrates that carbon oxidation proceeds more stably in neat internal olefin combustion systems, the radical-inhibiting effect of NH_3_ can only manifest after prolonged simulation via O_2_ depletion and insufficient OH supply.

In Figure 4c, the CO evolution curves of neat isobutene combustion and NH_3_-doped isobutene combustion nearly overlap, and the discrepancy in CO mole numbers at 4000 ps is marginal. Meanwhile, CO_2_ concentrations under NH_3_ doping are only slightly reduced relative to the neat condition. These observations indicate that neat isobutene combustion inherently sustains a high-intensity radical pool, where the radical competitive suppression effect and radical replenishment effect induced by NH_3_ nearly counteract each other.

In contrast to the three butene isomers, CO formation is persistently suppressed when butane is blended with NH_3_. For n-butane doped with NH_3_, CO concentrations are maintained at lower levels than the neat n-butane case over the whole simulation period. In terms of CO_2_, its concentration under NH_3_ addition is lower than that of neat combustion within 0~2500 ps, the concentration difference narrows and fluctuates alternately from 2500 ps to 3200 ps. Once the simulation time exceeds 3200 ps, CO_2_ concentrations in the NH_3_-doped system decline continuously, whereas CO_2_ yields in neat n-butane combustion keep increasing steadily. For isobutene blended with NH_3_, both CO and CO_2_ concentrations are persistently lower than those of neat isobutane combustion, and CO_2_ formation undergoes stable inhibition throughout the simulation. This reveals that NH_3_ preferentially consumes oxidative radicals such as OH and O during isobutane combustion, which impedes the full oxidation of carbon-containing intermediates into CO and further restrains the subsequent conversion of CO to CO_2_.

**Figure 5:**
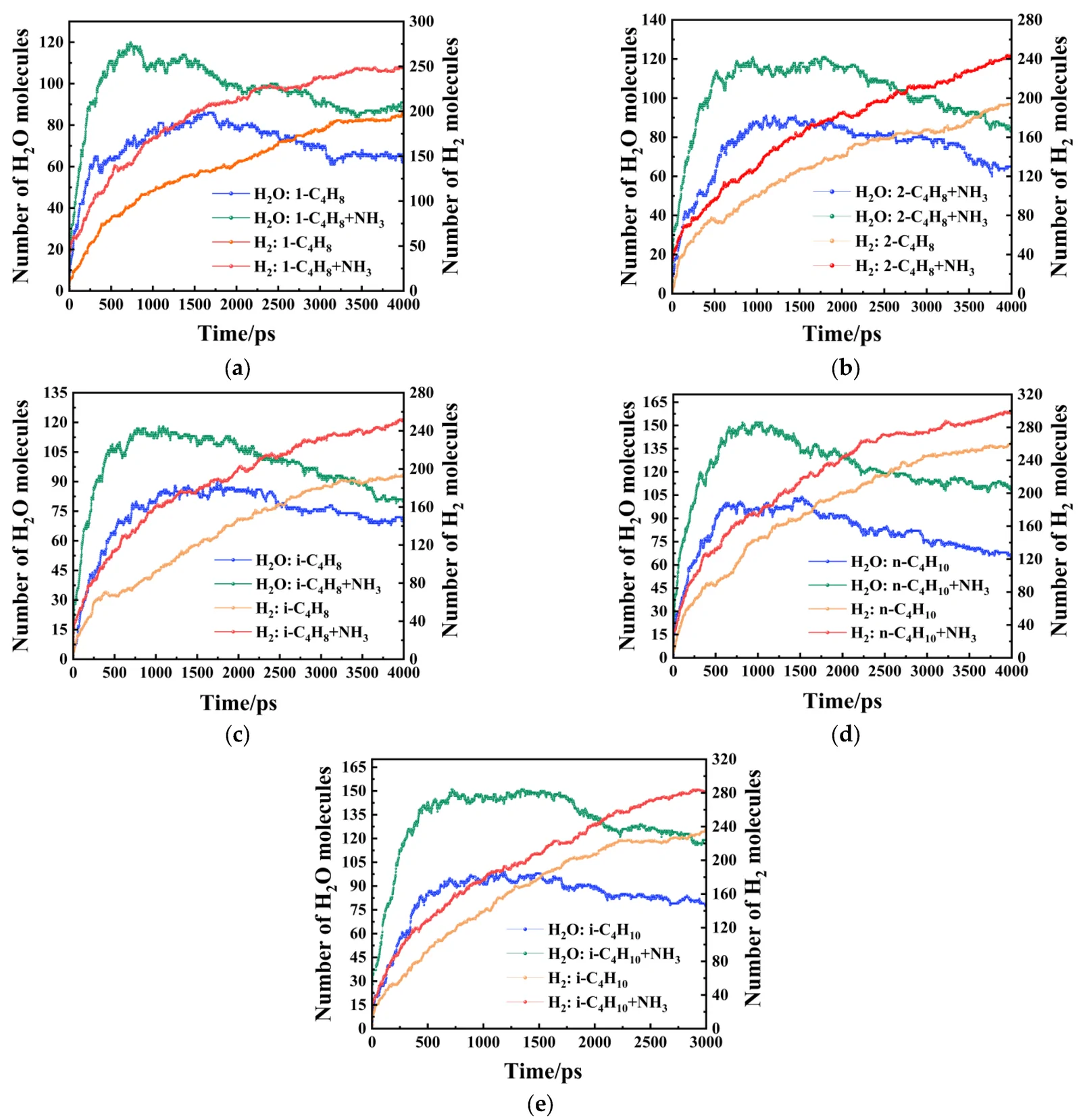
Temporal evolution of H_2_O and H_2_ during the combustion of C_4_ isomers blended with NH_3_: (**a**) 1-butene, (**b**) 2-butene, (**c**) isobutene, (**d**) n-butane, (**e**) isobutane.

NH_3_ can serve as an additional hydrogen source to facilitate the generation of H_2_O and H_2_. Figure 5 illustrates the temporal evolution profiles of H_2_O and H_2_ during the combustion of C_4_ isomers blended with NH_3_. Under all NH_3_-blended conditions, the molecular count of H_2_O exceeds that under pure-fuel combustion at corresponding time intervals. In all cases, the H_2_O concentration rises rapidly at first, reaches a peak, and then decays slowly. In Figure 5a, for the 1-butene/NH_3_ blended case, the peak molecular number of H_2_O is approximately 120 at a peak time of around 500 ps. For pure 1-butene combustion, the peak value is roughly 85 molecules, with the peak delayed to 1500 ps. As shown in Figure 5b,c, the H_2_O peaks of 2-butene and isobutene with NH_3_ addition occur within 500~1000 ps, with peak molecular counts of about 120 and 118, respectively. Both values are higher than the corresponding pure-fuel peaks of 90 and 88 molecules. Figure 5d,e correspond to n-butane and isobutane mixed with NH_3_, which exhibit even higher H_2_O peak quantities of approximately 150 molecules, markedly surpassing the pure-fuel peak values of 105 and 95 molecules, respectively.

The elevated production of H_2_O originates from the products formed via the reaction between NH_3_ and OH, as well as oxidation chain reactions involving NH_3_. Once O_2_ is fully consumed, the formation rate of OH decreases, and the generated H_2_O is subsequently consumed by carbon-rich species to produce CO and H_2_. Accordingly, NH_3_ addition yields a higher peak concentration of H_2_O accompanied by a more rapid post-peak decay, which correlates well with the mid-to-late-stage accumulation of CO and the continuous rise in H_2_ concentration. In contrast, the evolution trend of H_2_ follows a consistent pattern across all test cases. The molecular population of H_2_ under NH_3_-blended combustion of C_4_ isomers is universally higher than that under pure fuel combustion. Additionally, H_2_ exhibits no distinct peak and undergoes continuous growth throughout the entire reaction duration. This phenomenon fundamentally arises from the successive dehydrogenation of NH_3_, which supplies supplementary H for the overall reaction system, and the subsequent recombination of H to form H_2_. Furthermore, NH_3_ and its decomposition intermediates preferentially react with OH, reducing the likelihood of H_2_ being scavenged by OH and thereby further facilitating the continuous accumulation of H_2_.

Therefore, in terms of reaction products, NH_3_ blending significantly alters the oxidation level of the reaction system. On the one hand, the formation of CO_2_ is suppressed. On the other hand, the yields of H_2_ and H_2_O rise remarkably, enhancing the reductive nature of the combustion system. Such discrepancies reveal that the effects of NH_3_ addition on combustion product distribution stem not only from the hydrogen atoms inherent in NH_3_ itself but also from the molecular structure of the parent C_4_ fuel.

**Figure 6:**
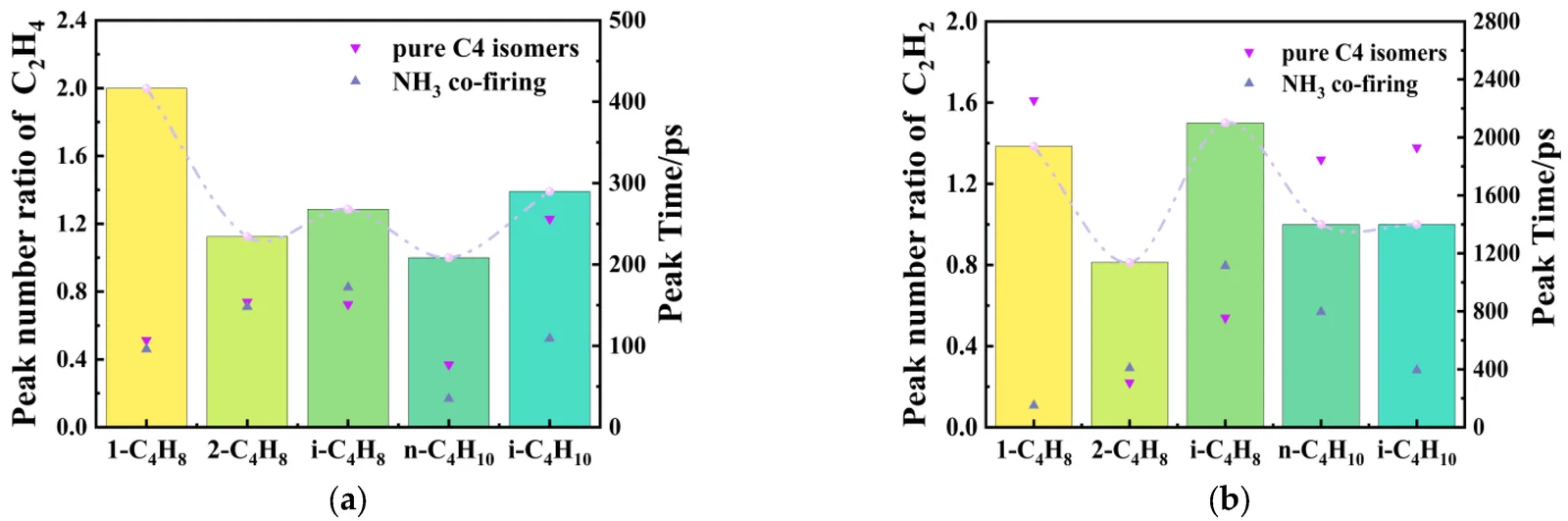
Ratios of molecular population and peak time for C_4_ isomers under pure-fuel combustion and NH_3_-blended combustion: (**a**) C_2_H_4_, (**b**) C_2_H_2_.

NH_3_ blending alters the peak concentrations of small-molecule pyrolysis products and triggers divergent C_2_/C_3_ reaction pathways [28,33]. C_2_H_4_, an important product from the β-scission of C_4_ fuels, acts as an essential precursor for acetylene C_2_H_4_. Figure 6a reveals that NH_3_ addition imposes varying degrees of suppression on the peak C_2_H_4_ concentration of different isomers. The inhibition effect is the most pronounced for 1-butene, followed by isobutane, isobutene and 2-butene, whereas n-butane is barely affected. This demonstrates that NH_3_ accelerates the dehydrogenation and oxidative consumption of C_2_H_4_ by increasing the concentrations of H, OH and other radicals. Figure 6b illustrates that the evolution of C_2_H_2_ displays selectivity. NH_3_ blending reduces the peak C_2_H_4_ values for 1-butene and isobutene, while elevating the C_2_H_4_ peak concentration of 2-butene. This is attributed to the enhanced dehydrogenation of small molecules driven by the higher H concentration induced by NH_3_, whereby the production rate of C_2_H_2_ outweighs its consumption rate. In comparison, the peak C_2_H_2_ concentrations of butanes show little sensitivity to NH_3_ addition. Accordingly, NH_3_ does not exert a universal inhibitory effect across all C_4_ isomers. Instead, differentiated impacts on individual isomers are achieved by modulating the competitive interplay among C_2_H_4_ dehydrogenation, oxidative consumption of C_2_H_2_, and fuel pyrolysis pathways.

**Figure 7:**
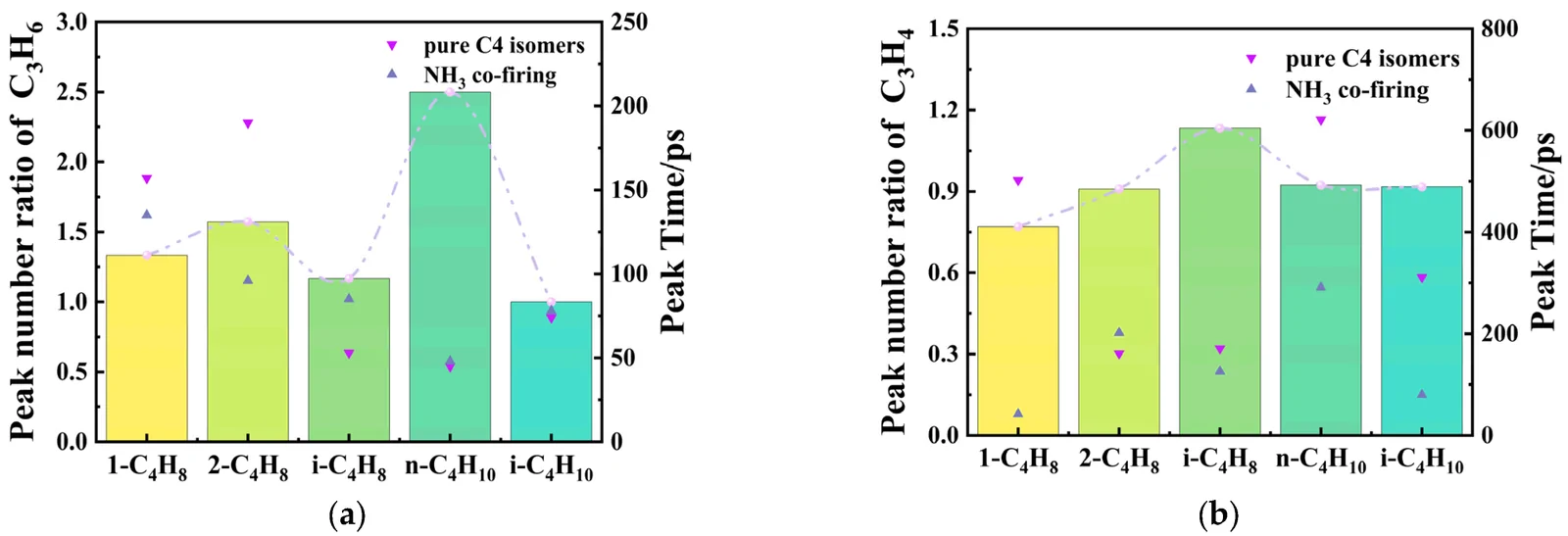
Ratios of molecular populations and peak occurrence times for C3 species under pure fuel combustion and NH_3_-blended combustion: (**a**) C_3_H_6_, (**b**) C_3_H_4_.

As illustrated in Figure 7, the peak concentration of C_3_H_6_ universally declines after NH_3_ addition. The strongest inhibitory effect is observed for n-butane, followed sequentially by 2-butene, 1-butene and isobutene, while n-butene exhibits nearly negligible variation. From the perspective of reaction pathways, C_3_H_6_ serves as the precursor of C_3_H_6_. Elevated concentrations of H and OH accelerate the dehydrogenation and oxidative consumption of C_3_H_6_, thereby lowering the C_3_H_6_ peak value for most C_4_ isomers. With the exception of isobutene, NH_3_ blending increases the peak C_3_H_4_ concentration of 1-butene, 2-butene, n-butane and isobutane, among which 1-butene presents the most remarkable enhancement. This indicates that for the C_3_ reaction pathway, the increased H concentration induced by NH_3_ facilitates successive dehydrogenation of C_3_H_6_ to produce C_3_H_4_. Nevertheless, the peak C_3_H_4_ concentration may also decrease if the parent fuel generates a high yield of C_3_ precursors, accompanied by intensified oxidative consumption via OH and O.

**Figure 8:**
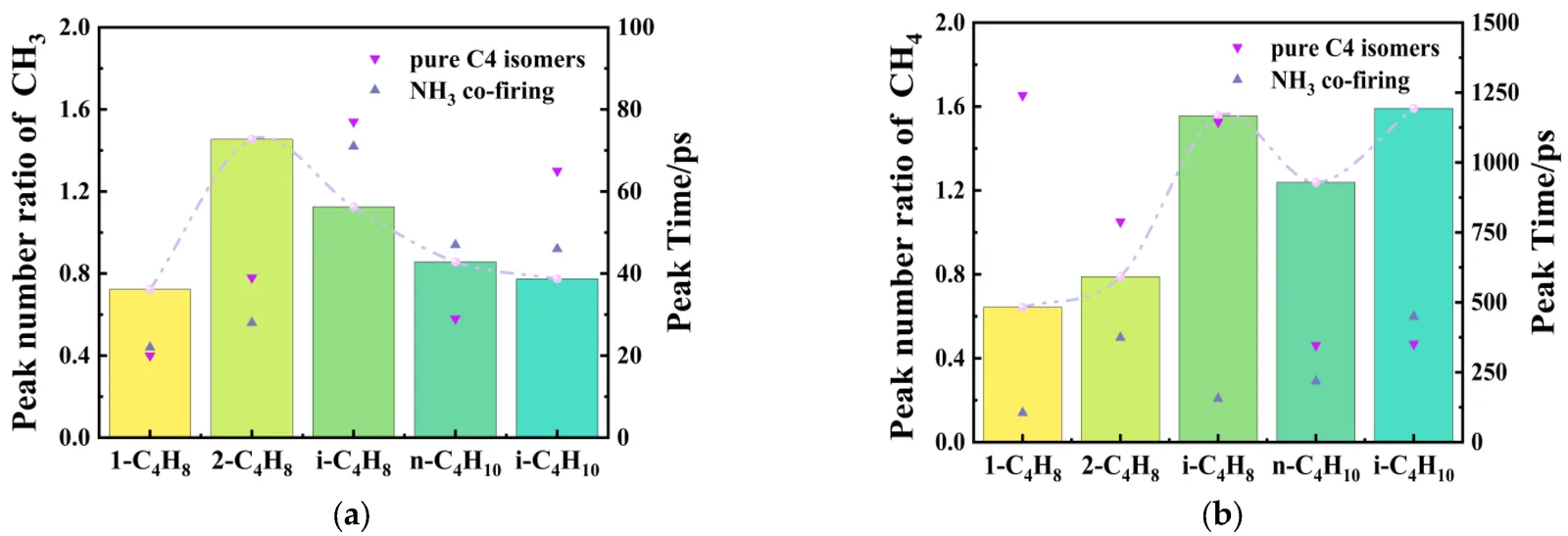
Ratios of molecular populations and peak occurrence times for C1 species under pure-fuel combustion and NH_3_-blended combustion: (**a**) CH_3_, (**b**) CH_4_.

Variations in C1 products can reflect the dominant pyrolysis pathways of the reaction system. As depicted in Figure 8, NH_3_ exerts disparate effects on the peak concentrations of CH_3_ and CH_4_. The peak CH_3_ concentration rises for isobutane, 1-butene and n-butane, whereas it is suppressed for 2-butene and isobutene. In contrast, the peak CH_4_ concentration is enhanced for 1-butene and 2-butene but inhibited for isobutane, isobutene and n-butane. CH_4_ is primarily formed via the recombination of CH_3_ and H. For 1-butene blended with CH_3_, both CH_3_ and H concentrations increase simultaneously, which accounts for the most prominent rise in the CH_4_ peak value. Isobutane and isobutene inherently produce abundant CH_3_. After NH_3_ addition, the elevated concentration of OH accelerates the dehydrogenation and consumption of CH_4_, resulting in a reduction in its peak concentration.

Overall, NH_3_ exerts dual regulatory effects on the combustion of C_4_ isomers. On the one hand, NH_3_ supplies H to promote fuel pyrolysis and dehydrogenation reactions. On the other hand, NH_3_ modulates the oxidative consumption of carbon intermediates through OH and O. Consequently, the effects of NH_3_ blending on the pyrolysis pathways of individual C_4_ isomers are summarized as follows. For 1-butene, the dominant pyrolysis pathway shifts from C_2_-centered pathways to C_1_- and C_3_-centered pathways. In the case of 2-butene, the yields of highly unsaturated C_2_ products increase, while the formation of C_3_H_6_ is suppressed. For isobutene, the generation of most soot precursors is inhibited. As for n-butane and isobutane, NH_3_ mainly alters the dynamic equilibrium between saturated and unsaturated C3 intermediates.

These trends can be quantitatively compared with published experimental and modeling studies to confirm their physical robustness. Regarding the suppression of CO_2_, NH_3_ is a carbon-free fuel, so blending NH_3_ directly dilutes the carbon source of the system, and the reduction in CO_2_ emissions with increasing NH_3_ blending ratio is a general rule in the ammonia co-firing community. For instance, Li et al. [34] carried out three-dimensional pore-scale numerical simulations of NH_3_-CH_4_ premixed combustion in a double-layer spherical stacked porous medium burner and found that CO_2_ emissions reach a peak at an NH_3_ blending ratio of approximately 10%, decline significantly once the NH_3_ concentration exceeds 20%, and reach the lowest level at 30%, with CO_2_ formation being further suppressed under fuel-rich conditions (*φ* > 1). This is consistent with the present results: under the strongly fuel-rich condition of *φ* = 4, the CO_2_ yield of every C_4_ isomer system blended with NH_3_ remains lower than that of the corresponding neat combustion throughout the whole reaction process, in which the CO_2_ formation of the isobutane system is stably suppressed over the entire simulation time, and the CO_2_ yield of the n-butane system keeps declining after 3200 ps while that of the neat case still rises. Regarding the promotion of H_2_ and H_2_O, NH_3_ is a hydrogen-rich, carbon-free fuel that can decompose into H_2_ and N_2_ at elevated temperatures, and its oxidation reactions mainly produce H_2_O and N_2_. Under fuel-rich (oxygen-lean) conditions, the pyrolysis and partial oxidation of NH_3_ supply abundant H to the system and promote H_2_ accumulation, which agrees with the conclusion that “NH_3_ promotes H generation” in the porous-medium combustion study [34] and with the summary of hydrogen-rich fuel combustion characteristics in the NH_3_ combustion review. Quantitatively, the peak H_2_O concentrations of all systems increase markedly after NH_3_ blending (e.g., from 85 to 120 molecules for 1-butene with the peak time advanced from 1500 ps to 500 ps, and from 105 to 150 molecules for n-butane), and the final H_2_ yields also increase significantly, consistent with the mechanisms reported in the above literature. Furthermore, our group has also observed the inhibitory effects of NH_3_ on CO_2_ formation and soot precursors as well as the promotion of H_2_/H_2_O in C_4_H_10_ combustion with H_2_-NH_3_ addition [32], NH_3_-doped ethylene flames [16], and ethylene/hydrogen/ammonia laminar diffusion flames [7] using the same ReaxFF force field, indicating that the present trends are consistent across systems and settings rather than artifacts of the force field or simulation setup.

### 2.4. Soot Formation Mechanism of NH_3_-Co-Fired C_4_ Isomers

Comparisons of soot microstructures help further clarify the effects of isomer configurations and NH_3_ blending on soot formation. Table 1 illustrates the microstructure, molecular formula, C/H atomic ratio and internal benzene ring number of the maximum soot particles generated within the simulation cycle under five neat combustion conditions, including pure 1-butene, 2-butene, isobutene, n-butane and isobutane flames.

Overall, the number of benzene rings in soot produced by butene fuels is higher than that in soot generated from butane fuels. Among all tested conditions, the flame fueled with 1-butene yields soot containing the maximum benzene ring count of 18, followed by isobutene with 17 benzene rings and 2-butene with 14 benzene rings. In contrast, soot formed under n-butane and isobutane flames only contains 2 and 3 benzene rings respectively, which are considerably lower than those observed for butene flames. This result demonstrates that butene fuels are more prone to form aromatic ring structures and accelerate soot growth under fuel-rich and high-temperature environments. Such discrepancy originates from the distinct molecular configurations of the fuels. Butenes contain C=C double bonds, during high-temperature pyrolysis and dehydrogenation, they can directly produce unsaturated active intermediates including allyl species, alkynes, and propargyl radicals. These intermediates rapidly form benzene rings and PAH structures via addition, cyclization and condensation reactions, thereby supplying abundant precursors for soot inception. By contrast, butanes have no carbon–carbon double bonds. They must undergo sequential dehydrogenation, bond scission and radical recombination reactions to generate unsaturated small molecules and aromatic precursors.

The C/H atomic ratio reflects the maturity of soot particles. In general, a higher C/H ratio corresponds to soot structures with higher carbon abundance and lower hydrogen content. By contrast, a lower C/H ratio indicates limited aromatic condensation and dehydrogenation of soot [33,35]. The flame fueled with isobutene exhibits the maximum C/H ratio, revealing a high carbon enrichment level of its soot molecules, followed by 1-butene and then 2-butene. The C/H ratios under butane combustion conditions are remarkably lower, which implies that the soot molecules generated within the present simulation cycle retain abundant H, accompanied by low aromatization degree and structural maturity. Combined analysis of the C/H atomic ratio and the number of six-membered rings demonstrates that soot from butene flames possesses not only more benzene rings but also generally higher C/H ratios compared with butane-derived soot. This evidence proves that butene fuels readily undergo dehydrogenation, cyclization and aromatic condensation reactions under high-temperature and fuel-rich conditions, thereby forming carbon-enriched microstructures that are closer to mature soot. The above conclusions are consistent with the microstructural configurations of maximum soot particles generated by the combustion of various C_4_ isomers displayed in Table 1.

Table 2 presents the microstructure, molecular formula, C/H atomic ratio and internal benzene ring count of the maximum soot particles produced within the simulation cycle for NH_3_-blended flames of 1-butene, 2-butene, isobutene, n-butane and isobutane. Compared with Table 1, the C/H atomic ratios of all test cases rise after NH_3_ blending. This phenomenon reveals that NH_3_ addition alters the reaction pathways during soot growth. The elevated C/H ratio indicates an overall carbon-enriched tendency of soot molecules. Nevertheless, nitrogen originating from NH_3_ can embed into the molecular skeleton of soot, disrupt the cyclization reaction for aromatic carbon structures, and further modify the evolutionary characteristics of soot aromatic skeletons.

The N/C atomic ratio equals zero for all neat combustion cases, while this ratio increases remarkably under NH_3_-blended conditions, demonstrating that N can be incorporated into the molecular structure of soot after NH_3_ participates in combustion reactions [36]. The N doping degree varies with different fuels. Among all test flames, the NH_3_-blended isobutane flame achieves the highest N/C ratio, followed by n-butane, which indicates stronger N doping effects for NH_3_-blended butane fuels. The N/C ratios of butene flames are relatively low, yet these values still verify the involvement of nitrogen species in soot formation. Mechanistically, NH_3_ decomposes into nitrogen-containing active radicals such as NH_2_, NH and N under high-temperature environments. These nitrogenous species can react with hydrocarbon radicals, aromatic radicals or active sites on soot surfaces to form C-N bonds, nitrogen heterocycles and other nitrogen-containing structures. On the one hand, NH_3_ blending modifies the elemental composition of soot molecules. On the other hand, it hinders the continuous formation of aromatic carbon networks and further regulates the graphitization degree of soot particles.

Analysis based on the number of six-membered rings reveals that the quantity of six-membered rings under NH_3_-blended conditions is larger than that of the corresponding neat fuel flames, with a particularly prominent increment observed for butane systems. Soot generated from neat butene flames already contains abundant six-membered rings. After NH_3_ blending, the number of six-membered rings rises slightly, accompanied by simultaneous increases in C/H and N/C atomic ratios. This result indicates that N is incorporated into soot molecules during soot growth. It can thus be inferred that NH_3_ blending fails to suppress the formation of six-membered rings, whereas N doping may reduce the ordering degree of aromatic structures. For alkane flames, the increased yield of six-membered rings cannot be fully attributed to the direct promotion effect of NH_3_. Within the identical simulation duration, soot produced by neat alkane combustion remains at the early growth stage. In contrast, NH_3_ blending modifies reaction pathways, radical pools and nitrogen-containing intermediates, thereby generating more nitrogen-doped soot structures.

## 3. Methods

### 3.1. Operating Conditions

Combustion is a coupled process involving chemical bond cleavage, recombination and free-radical chain reactions, whose transient atomic-scale evolution is difficult to track via experimental approaches. After years of development, ReaxFF MD enables accurate atomic-level characterization of bond breaking and formation throughout reactions, and has emerged as a core simulation technique for elucidating microscale reaction mechanisms [37,38,39]. The theoretical foundation of ReaxFF MD rests on bond-order theory, consisting of a series of bonded and non-bonded energy terms. In practical calculations, these energy terms are expressed as potential function formulas, alongside empirical parameters required for computation—including bond orders, bonded terms and non-bonded terms between all known element pairs derived from quantum mechanical calculations—stored within the force field parameter set. The functional relationship between the total system potential energy (*E_system_*) and the potential functions of individual energy terms is given in Equation (1).

(1) $$E_{\mathrm{system}}={\;E}_{\mathrm{bond}}+E_{\mathrm{over}}+E_{\mathrm{under}}+E_{\mathrm{val}}+E_{\mathrm{pen}}+E_{\mathrm{coa}}+E_{\mathrm{tors}}+E_{\mathrm{conj}}+E_{\mathrm{vdWasls}}+E_{\mathrm{Coulomb}}$$

In the above equation, *E_system_* denotes the total potential energy of the simulation system. Specifically, *E_bond_* represents bond energy, *E_over_* and *E_under_* correspond to the coordination correction energies for over-coordination and under-coordination, respectively. *E_val_* is the valence angle energy, *E_pen_* the penalty energy, *E_coa_* the three-body conjugation energy, *E_tors_* the torsion angle energy, and *E_conj_* the four-body conjugation energy. The aforementioned eight terms collectively constitute the bonded interaction energy component. The non-bonded interaction energy consists of two contributions: *E_vdWaals_* for van der Waals interactions and *E_Coulomb_* for Coulombic interactions.

Table 3 summarizes all simulation cases adopted in this work, which were designed following the single-variable control principle. Five typical C_4_ isomers, namely 1-butene, 2-butene, isobutene, n-butane and isobutane, were selected as research subjects. Two series of simulation cases were established, including baseline groups of pure fuels and comparative groups with NH_3_ blending, resulting in a total of 10 simulation cases. The initial *T* was fixed at 2800 K and the *ρ* was set to 0.3 g/cm^3^, with a fuel-rich *φ* of 4 [40,41]. The number of O_2_ was calculated according to the stoichiometric ratio.

It should be noted that ReaxFF MD is a molecular-scale “computational experiment” method aimed at revealing reaction pathways and mechanisms at the atomic level, rather than reproducing the macroscopic operating conditions of real combustion devices. By accelerating reactions at elevated temperatures, ReaxFF enables chemical processes that occur on micro- to millisecond timescales to be fully observed within a nanosecond simulation window. This “high-temperature acceleration” strategy is a common practice in ReaxFF reactive-dynamics studies [37,38]. The rationality of the simulation conditions adopted here is as follows: T = 2800 K lies within the adiabatic flame temperature range of hydrocarbon combustion, providing sufficient activation energy for key processes such as C_4_ isomer decomposition, oxidation and soot-precursor formation; the high system density of 0.3 g/cm^3^ increases molecular collision frequency and accelerates reaction progress, thereby yielding sufficient reaction events and statistical samples within limited computational resources; the strongly fuel-rich equivalence ratio *φ* = 4 is consistent with the physical conditions of soot formation, ensuring that each system generates sufficient and distinguishable soot structures within the simulation window; and the 4000 ps simulation duration guarantees the statistical significance of comparisons among different systems. Meanwhile, ReaxFF itself possesses inherent limitations: (i) it describes bond formation and cleavage based on an empirical bond-order potential, and its reaction enthalpies and energy barriers are less accurate than those of ab initio methods and detailed chemical kinetic mechanisms [38], so this work does not rely on absolute thermochemical quantities and only compares relative trends between systems; (ii) pressure is not an independent control variable in the NVT ensemble, making it difficult to capture third-body collision and pressure-dependent unimolecular dissociation effects, so pressure effects are not addressed here; (iii) at 2800 K the reactions proceed predominantly along high-temperature pathways, which may differ from those at low/intermediate temperatures, and therefore the conclusions of this work are limited to high-temperature fuel-rich conditions; (iv) the simulation system contains only hundreds of C_4_ molecules (thousands of atoms), and within the 4000 ps timescale soot particles are only in the early nucleation and molecular-growth stage, without covering the complete lifecycle of surface growth, coalescence and oxidation of real soot particles, which limits the statistical representativeness of soot-related conclusions.

### 3.2. Simulation Details

**Figure 9:**
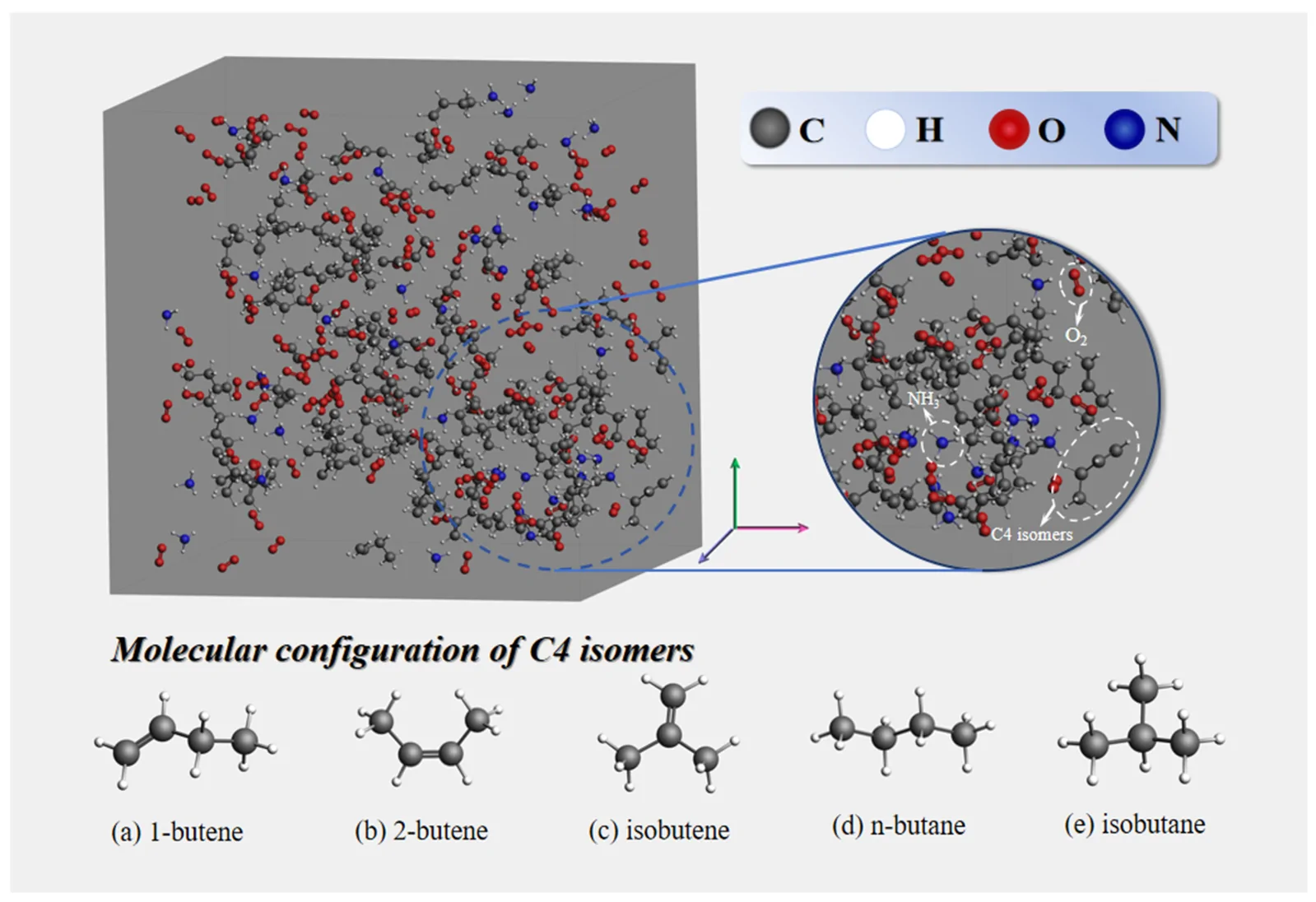
Optimized reaction ensemble and molecular configuration of C_4_ isomers.

The construction of the molecular system model was mainly implemented via the ReaxFF module embedded in the Amsterdam Density Functional (ADF) 2021.107 software package, where the CHON-2019 reactive force field was adopted with a time step of 0.25 fs. To guarantee the stability of the reaction ensemble for simulating incomplete combustion of C_4_ isomers, geometry optimization and energy minimization were sequentially performed to achieve the minimum energy state and reasonable structural configuration of the initial system. Figure 9 presents the optimized ensemble and molecular structures of C_4_ fuels. It can be observed that fuels, oxidizers and additives are homogeneously mixed after optimization, approximating the state of a premixed flame and facilitating subsequent kinetic analysis.

All simulation cases in this work adopted the NVT ensemble, where the number of atoms, system volume and temperature were kept constant. The Berendsen thermostat (Intel Xeon E5-2650 V4, Intel Corporation, Santa Clara, CA, USA) with a damping coefficient of 100 fs was utilized for temperature regulation. Trajectory analysis and statistical calculations of products and intermediates were conducted with a bond-order cutoff value of 0.3 Å. Periodic boundary conditions were applied throughout all simulations to eliminate the interference of boundary effects on reaction evolution. All computational cases were performed on a high-performance server equipped with 24 CPU cores (E5-2650 V4). A total simulation duration of 4000 ps via ReaxFF reactive molecular dynamics required a wall-clock time of 12 h. To ensure statistical reliability, three independent simulations with distinct initial configurations are performed for each operating condition, and the final data are averaged from the three independent ensemble-simulation runs. In the study of soot formation, it is generally accepted that the collision-induced nucleation of two tetracyclic aromatic pyrene molecules A4 gives rise to freshly nucleated soot particles containing 32 carbon atoms [42,43]. Therefore, in ReaxFF MD studies, as the reaction proceeds, a so-called young soot particle is considered to have formed once the number of carbon atoms within a given molecule is no less than 32. Furthermore, when the carbon-atom content of a soot particle is no less than 100, it is considered to have developed into a mature soot particle. These classification criteria have been adopted and validated multiple times in our previous studies [40,41]. Consequently, the same classification approach is retained in the present work to discuss the influence of ammonia doping on the soot-formation mechanism of C_4_ isomers.

Notably, although the present work does not include direct experimental calibration, the reliability of the ReaxFF calculations is supported by three lines of evidence. First, the CHON-2019 reactive force field employed here belongs to the ReaxFF empirical bond-order potential family whose parameters were fitted against quantum-mechanical calculations and has been widely validated in hydrocarbon oxidation, ammonia combustion and soot formation studies [37,38]. Second, the adopted soot-classification criterion (C_32_ as young soot and C_100_ as mature soot) follows the generally accepted PAH-collision nucleation framework [42,43] and has been repeatedly reproduced and validated in our previous ReaxFF studies under comparable conditions [40,41]. Third, the key trends observed in the present work—NH_3_ suppressing CO_2_ formation and soot-precursor generation while promoting H_2_/H_2_O yields—are qualitatively consistent with our group’s and other groups’ ReaxFF results obtained with the same force field across different fuel systems, including methane/ethylene combustion [40,41], C_4_H_10_ combustion with H_2_-NH_3_ addition [32], and ammonia-doped ethylene flames [7,16,36], as well as with experimental and modeling studies of NH_3_-CH_4_ combustion [23,24,34] and reviews of ammonia combustion [5]. This cross-system consistency indicates that the observed trends reflect robust physical chemistry rather than artifacts of the force field or simulation setup. Given the molecular-scale scope of this work, the calculations are used to reveal relative trends and reaction mechanisms rather than to reproduce absolute quantitative emission data, and no direct extrapolation to engineering devices is intended.

The present ReaxFF MD study involves several intentional assumptions and simplifications, which are stated explicitly below together with their justifications. (1) The NVT ensemble is adopted instead of an open-flow or constant-pressure configuration, because the objective is to isolate intrinsic chemical kinetics at a prescribed temperature rather than to reproduce heat-transfer or pressure variations; accordingly, pressure-dependent and third-body effects are not discussed in this work (see Section 3.1). (2) A homogeneous, perfectly premixed initial mixture is assumed. This simplification is appropriate for comparing intrinsic fuel reactivity under identical mixing conditions and deliberately excludes turbulence, fuel-droplet evaporation and non-premixed mixing, which would otherwise obscure the structural-comparative focus of this study. (3) Periodic boundary conditions are applied to mimic an infinitely large, homogeneous system and eliminate surface boundary effects; since only relative trends among the five C_4_ isomers are compared, finite-size effects influence all systems equally and do not bias the inter-isomer comparison. (4) A bond-order cutoff of 0.3 Å is used for species identification, which is a standard convention in ReaxFF trajectory analysis [38] and ensures that bonded/non-bonded states are defined consistently across all cases. (5) The simulation system contains only hundreds of fuel molecules (thousands of atoms) and evolves over 4000 ps. This finite size and time span are dictated by computational cost and are sufficient to capture fuel decomposition, oxidation and early soot nucleation, but they do not cover later soot growth, coalescence, oxidation or engineering timescales; consequently, soot-related conclusions are restricted to early-stage trends. (6) Only major product families—CO, CO_2_, H_2_, H_2_O, C_1_-C_3_ intermediates, PAHs and soot particles—are counted, while trace species and detailed NOx formation are outside the scope of this work. (7) Three independent runs with distinct initial configurations are averaged to control statistical noise. Together, these assumptions represent deliberate trade-offs that make atomic-scale mechanism resolution computationally affordable; because all five isomers and all NH_3_-blended cases are treated under identical settings, the comparative, mechanism-focused conclusions of this work are not affected by these simplifications.

To directly verify the accuracy of the molecular simulation method applied in this work, the key qualitative trends obtained from the ReaxFF calculations are compared against independent experimental observations on the same fuel families. First, the structural dependence of soot formation is reproduced: the present simulations show that neat olefins generate far more six-membered rings and soot precursors than alkanes, which is qualitatively consistent with counter-flow diffusion flame experiments [18], where the critical fuel concentrations for the macroscopic soot transition threshold follow the order 1-butene (9.0%) < isobutene (9.5%) < isobutane (19%) < n-butane (25%)—i.e., C_4_ olefins exhibit a far greater sooting tendency than their corresponding alkanes. Second, the inhibitory effect of NH_3_ on soot precursors observed in this work agrees with tube-furnace co-pyrolysis experiments of C_4_ isomers with NH_3_ at 1473 K [22], in which NH_3_ addition was found to reduce the concentrations of key soot precursors including C_2_H_2_, C_2_H_4_ and C_4_H_2_, and with experimental soot-morphology observations in ammonia-doped ethylene flames [8,16,36]. Third, the product trends induced by NH_3_ blending—suppressed CO_2_ formation and enhanced H_2_/H_2_O yields—are consistent with experimental and modeling studies of NH_3_-CH_4_ combustion [23,24,34] and with ammonia-combustion reviews [5]. Regarding the system size, each case contains 80 fuel molecules (hundreds of molecules and thousands of atoms in total), which lies within the typical range of ReaxFF combustion simulations reported in the literature [37,40,41] and is sufficient to resolve atomic-scale reaction pathways. Because all ten cases are compared under identical molecule counts, density and temperature, finite-size effects act equally on every system and do not bias the relative rankings among C_4_ isomers; statistical noise is further controlled by averaging three independent runs. In addition, all quantitative criteria used in this work—including the C_32_/C_100_ soot classification [42,43], the 0.3 Å bond-order cutoff [38], and the choice of T, ρ and φ—are grounded in published methods rather than arbitrary assumptions. Together, the qualitative agreement with independent experiments on the same fuel systems and the standard methodological criteria confirm that the molecular simulation method applied in this work is fit for its mechanistic, trend-comparison purpose; the conclusions are accordingly limited to relative trends and reaction pathways rather than absolute quantitative predictions.

## 4. Conclusions

In this work, ReaxFF MD simulations are performed at *T* = 2800 K and *φ* = 4 to investigate the pure combustion and NH_3_-blended combustion processes of five C_4_ isomers, namely n-butane, isobutane, 1-butene, 2-butene and isobutene. The main conclusions are summarized as follows:

(1) The combustion and soot formation of C_4_ isomers exhibit remarkable structural dependence. In the early reaction stage, the ignition rate is governed by the reactivity of terminal double bonds and tertiary C-H bonds. 1-butene and isobutane possess the fastest initial reaction rates, while 2-butene shows the slowest ignition due to steric hindrance. In the mid-stage of reactions, the cracking capacity of carbon skeletons dominates the reaction efficiency, and linear carbon skeletons deliver superior sustained reactivity. In the late combustion stage, resonance-stabilized allylic intermediates tend to persist in the system, hindering the oxidation of olefins. In terms of combustion products, 2-butene demonstrates the strongest oxidation capacity with the highest CO_2_ yield. Alkanes contain higher H, leading to notably greater H_2_ production compared with butenes.
(2) The regulation effect of NH_3_ doping on C_4_ isomers is structurally sensitive. For 1-butene and isobutane, NH_3_ only imposes transient inhibitory effects via radical competition, exerting limited influence on the total burnout time. By contrast, NH_3_ remarkably accelerates the cracking of 2-butene through supplementary H supply. NH_3_ addition drastically shortens the depletion time of O_2_, suppresses CO_2_ generation, and facilitates the accumulation of H_2_O and H_2_. Overall, NH_3_ reduces the oxidation level of the combustion system and enhances its reducibility. Furthermore, NH_3_ enriches the radical pool to alter hydrocarbon cracking pathways, resulting in differentiated evolution of small-molecule intermediates for various C_4_ isomers.
(3) With respect to soot formation, the quantity of six-membered rings for all C_4_ isomers rises under NH_3_-blended combustion relative to pure fuel combustion, among which n-butane achieves the most significant increment. Nevertheless, the elevated six-membered ring content of butanes cannot be fully attributed to the promotion effect of NH_3_. Under identical simulation durations, soot generated from pure alkanes remains in the early growth stage. NH_3_ doping accelerates the formation of nitrogen-containing soot structures by rearranging reaction pathways, modifying radical pools and regulating the evolution of nitrogen-bearing intermediates. N is incorporated into soot skeletons in the form of nitrogen functional groups and nitrogen heterocycles, simultaneously elevating the C/H and N/C atomic ratios. In addition, N doping fails to reduce the total yield of six-membered rings, yet it disturbs the construction of aromatic carbon frameworks and diminishes the ordering degree of aromatic structures within soot particles. It should be noted that the above conclusions represent mechanistic insights at the molecular level, supported by cross-system consistency with existing experimental and modeling studies, rather than direct quantitative calibration against engineering operating conditions.

## Figures and Tables

**Table 1:** The microstructure, molecular formula, C/H atomic ratio, and internal benzene ring quantity of the largest soot particles generated during the combustion of C_4_ isomers.

	(a) 1-C_4_H_8_	(b) 2-C_4_H_8_	(c) i-C_4_H_8_	(d) n-C_4_H_10_	(e) i-C_4_H_10_
Micro- structure	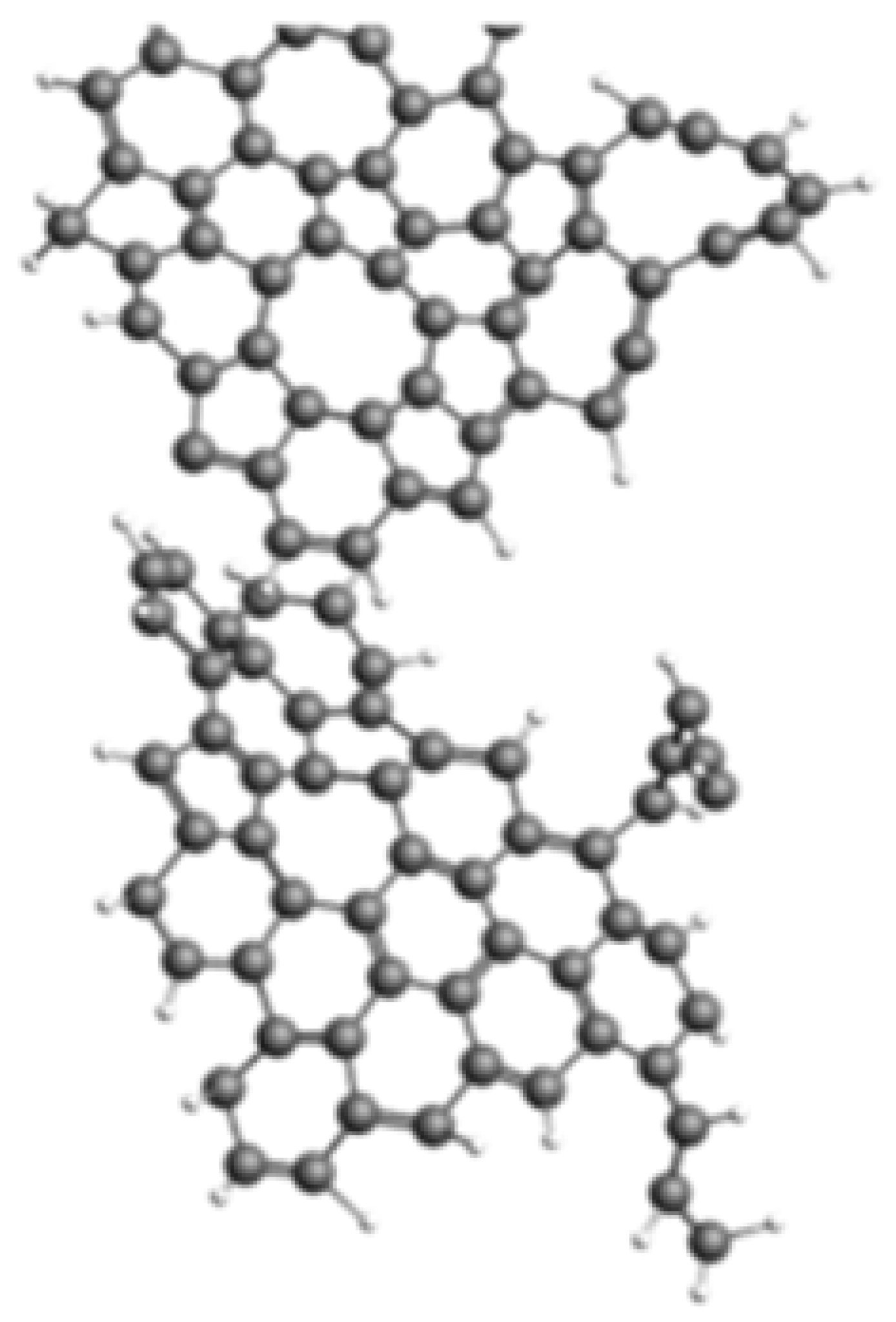	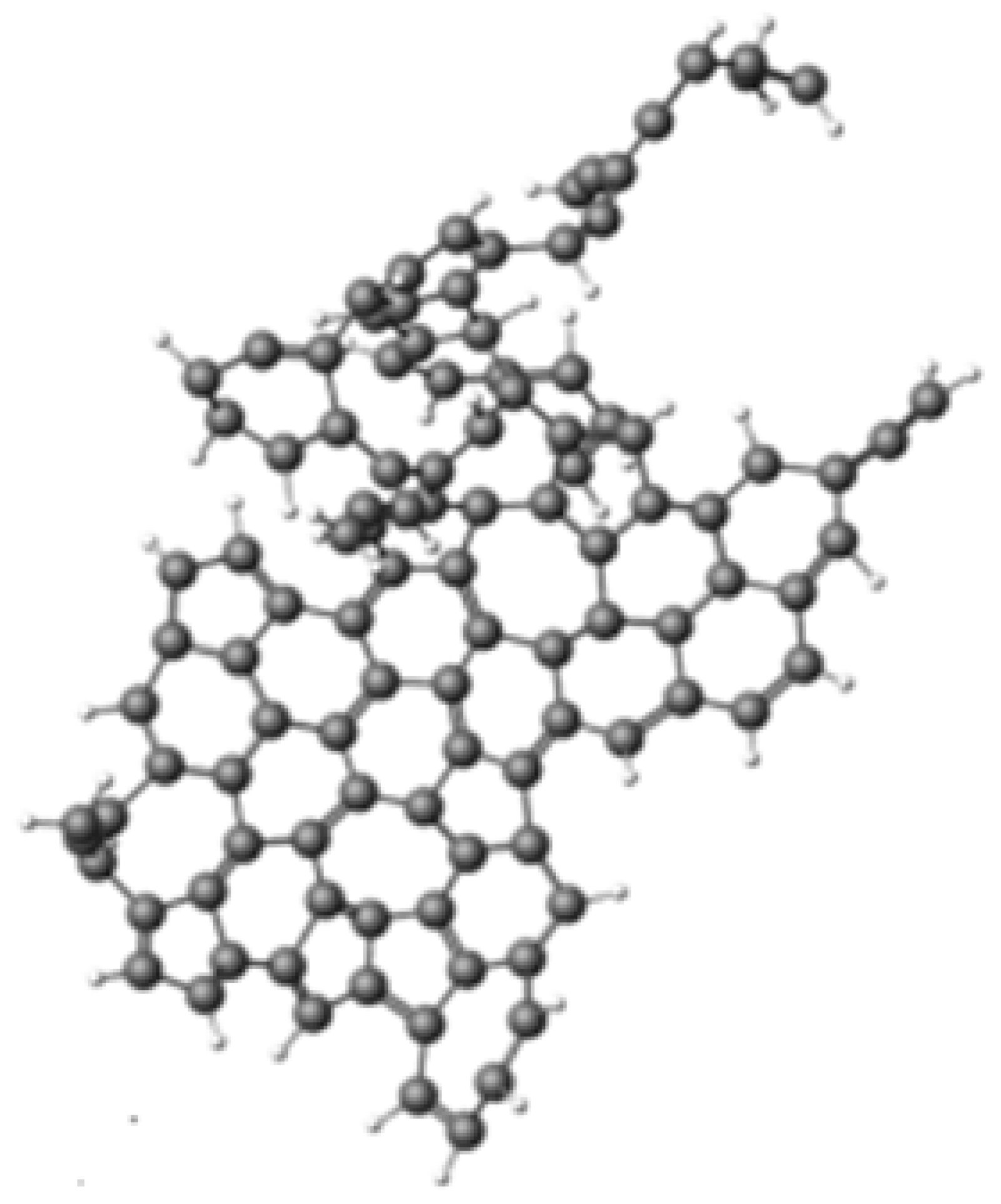	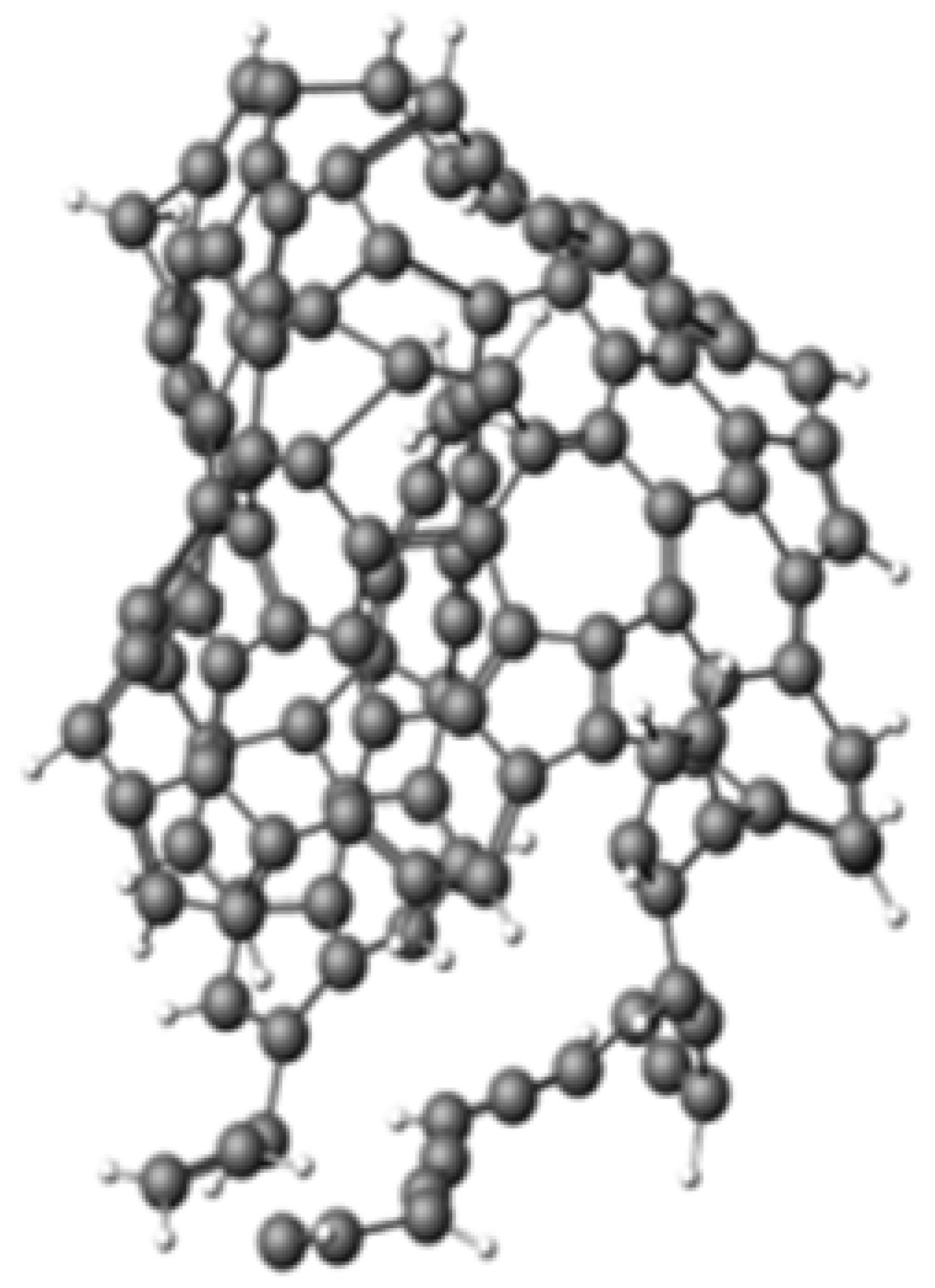	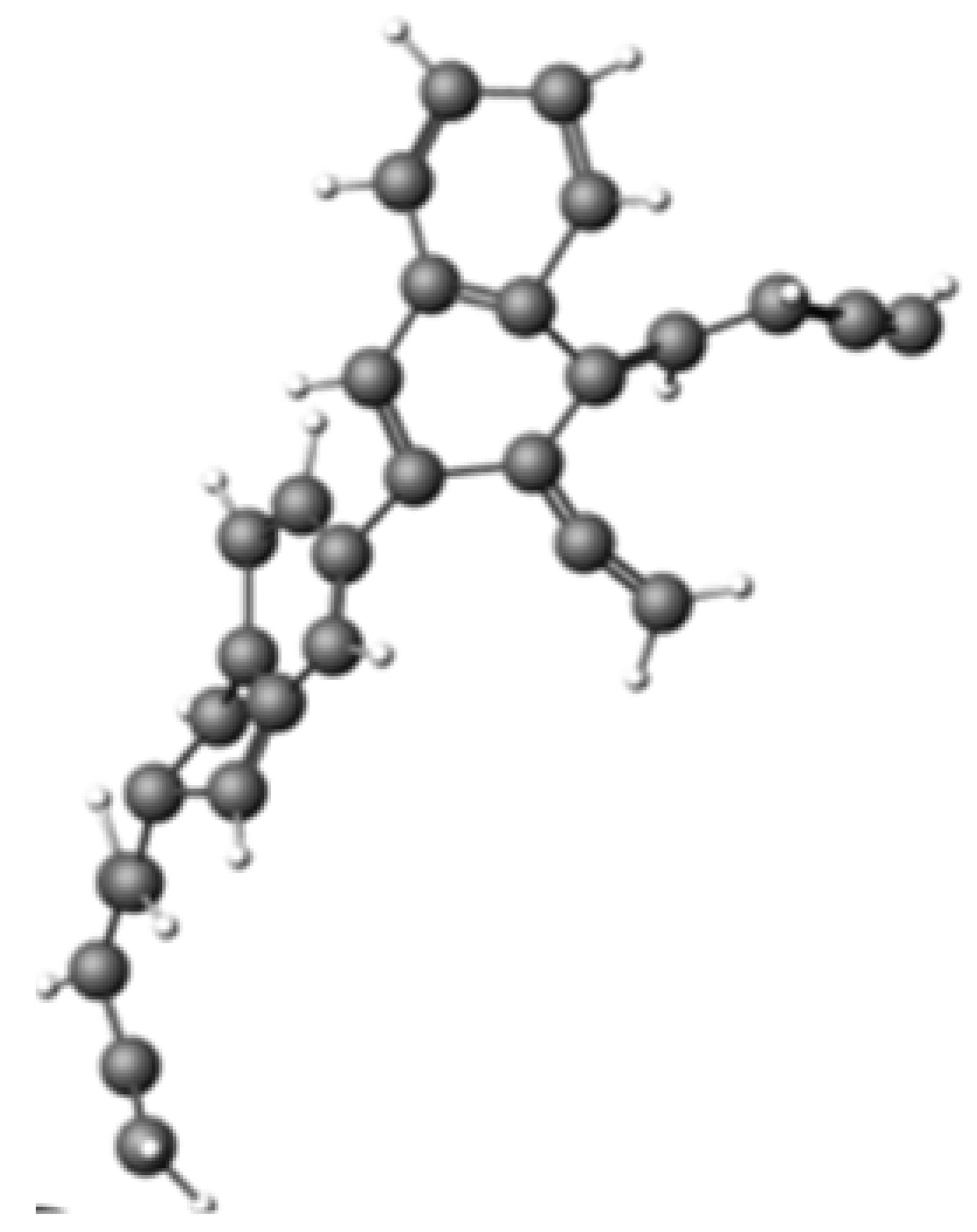	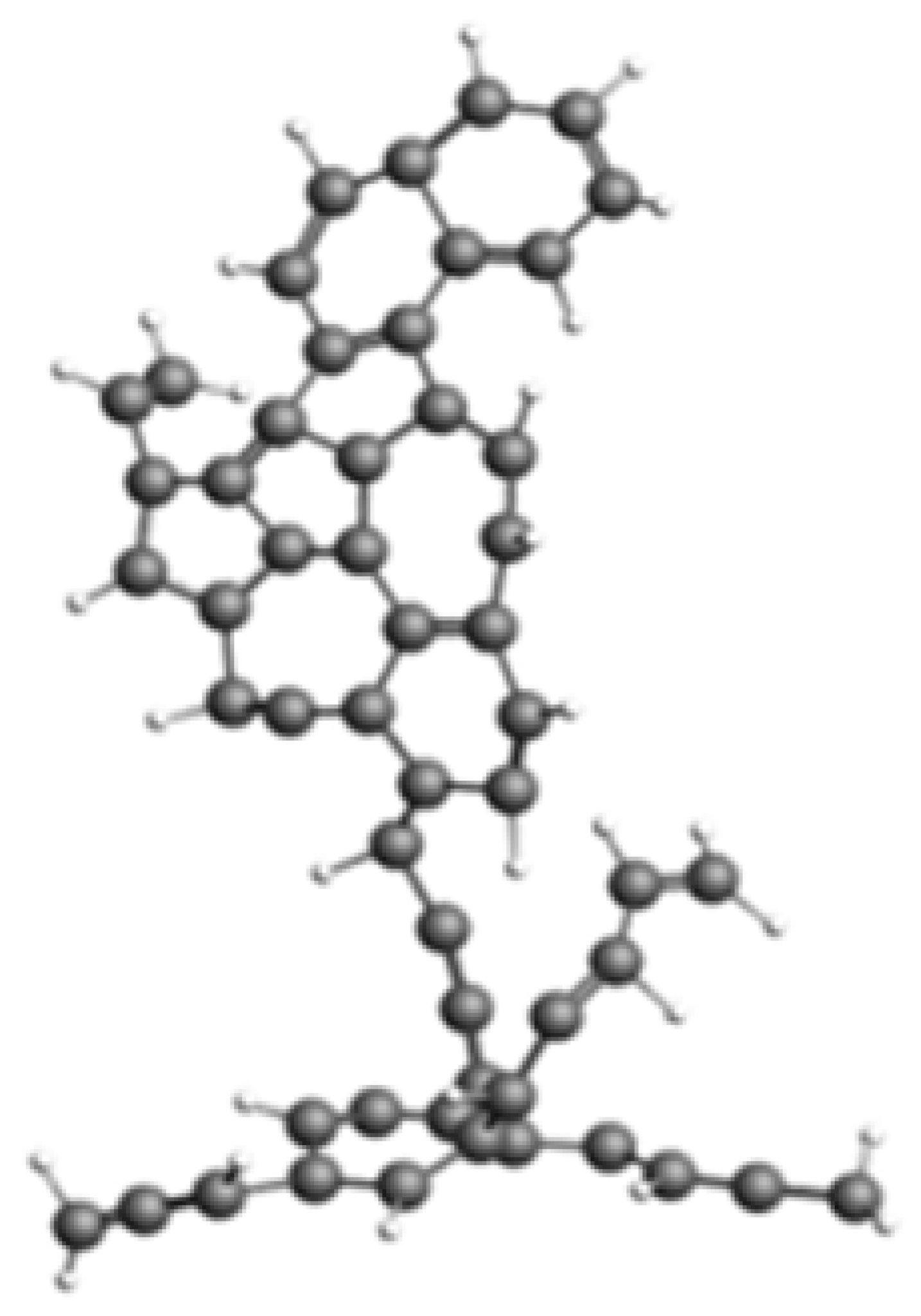
Molecular formula	C_108_H_39_	C_109_H_47_	C_127_H_42_	C_58_H_42_	C_54_H_31_
C/H atomic ratio	2.77	2.32	3.02	1.38	1.74
Number of benzene rings	18	14	17	2	2

**Table 2:** Morphology, molecular formula, C/H atomic ratio, N/C atomic ratio and internal benzene ring number of maximum soot particles generated during combustion of C_4_ isomers under NH_3_.

	(a) 1-C_4_H_8_	(b) 2-C_4_H_8_	(c) i-C_4_H_8_	(d) n-C_4_H_10_	(e) i-C_4_H_10_
Micro- structure	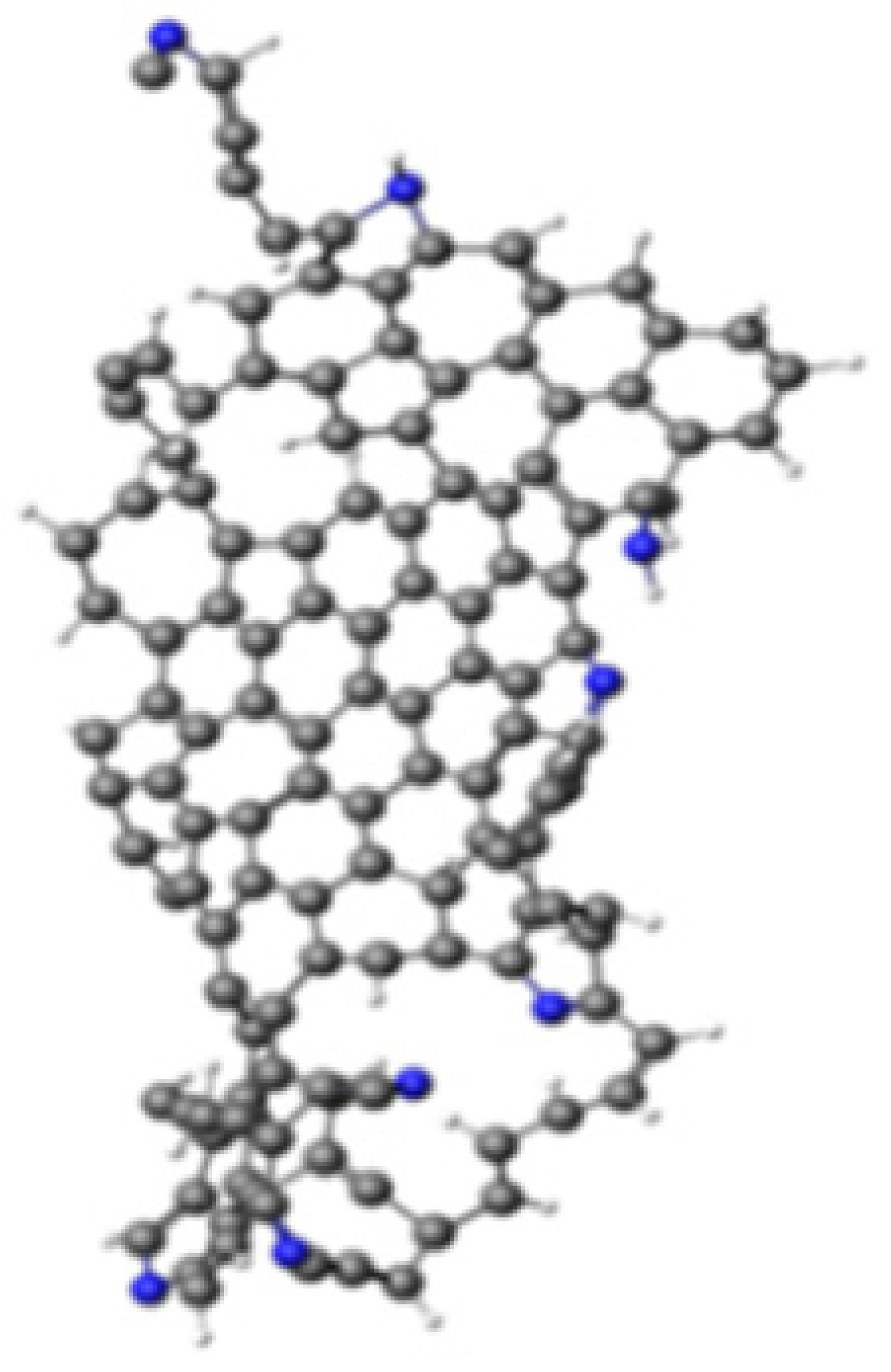	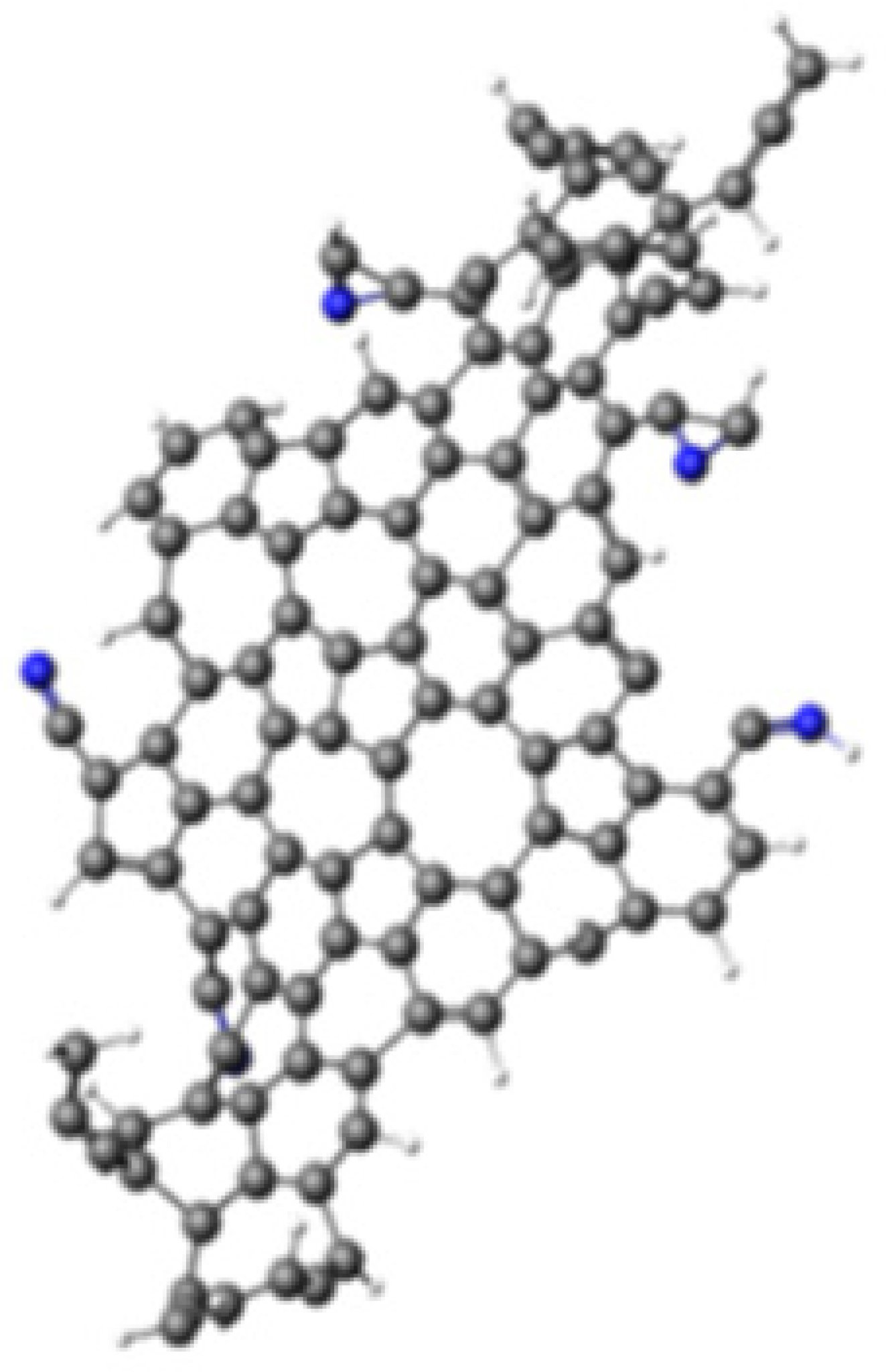	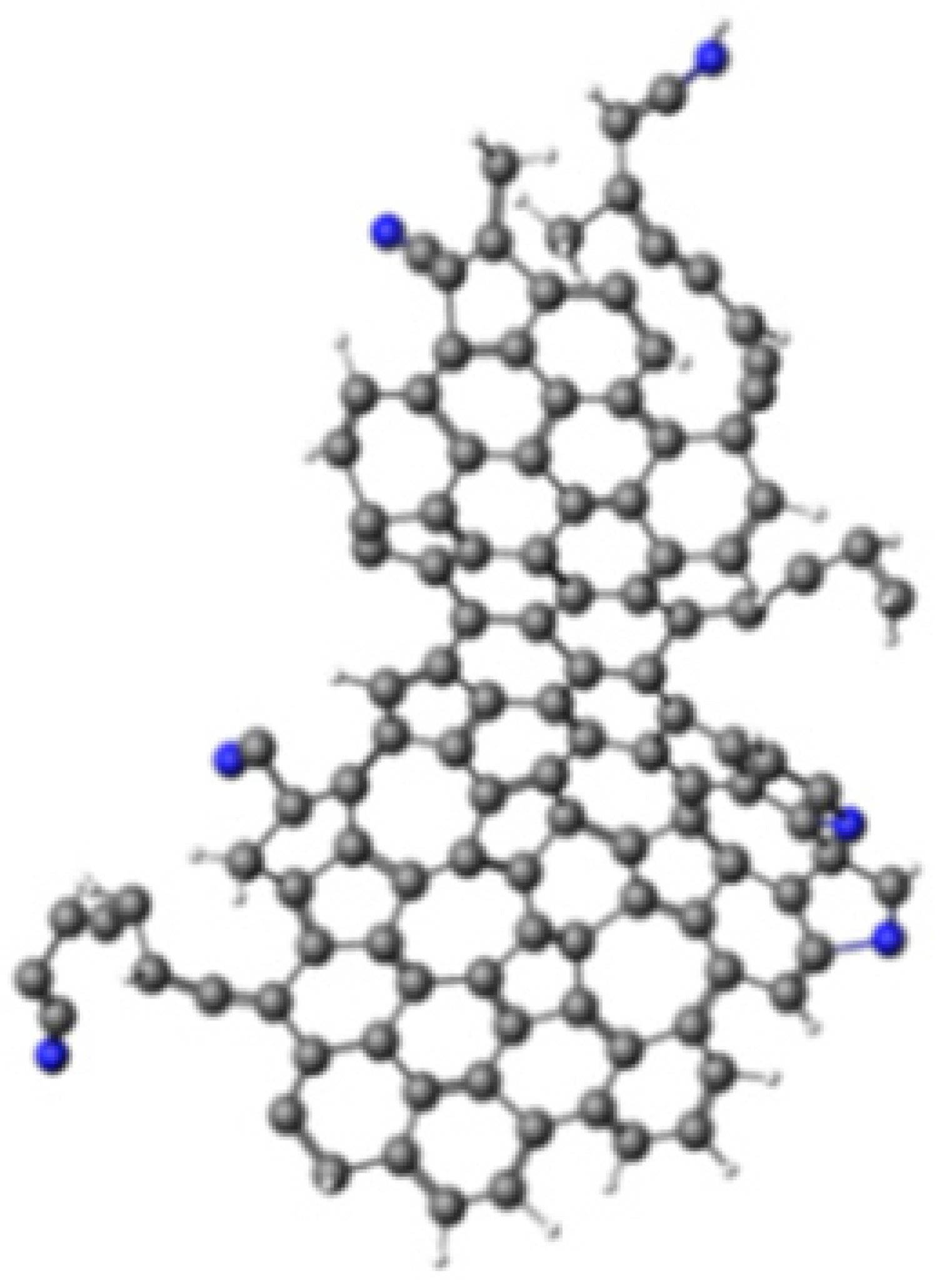	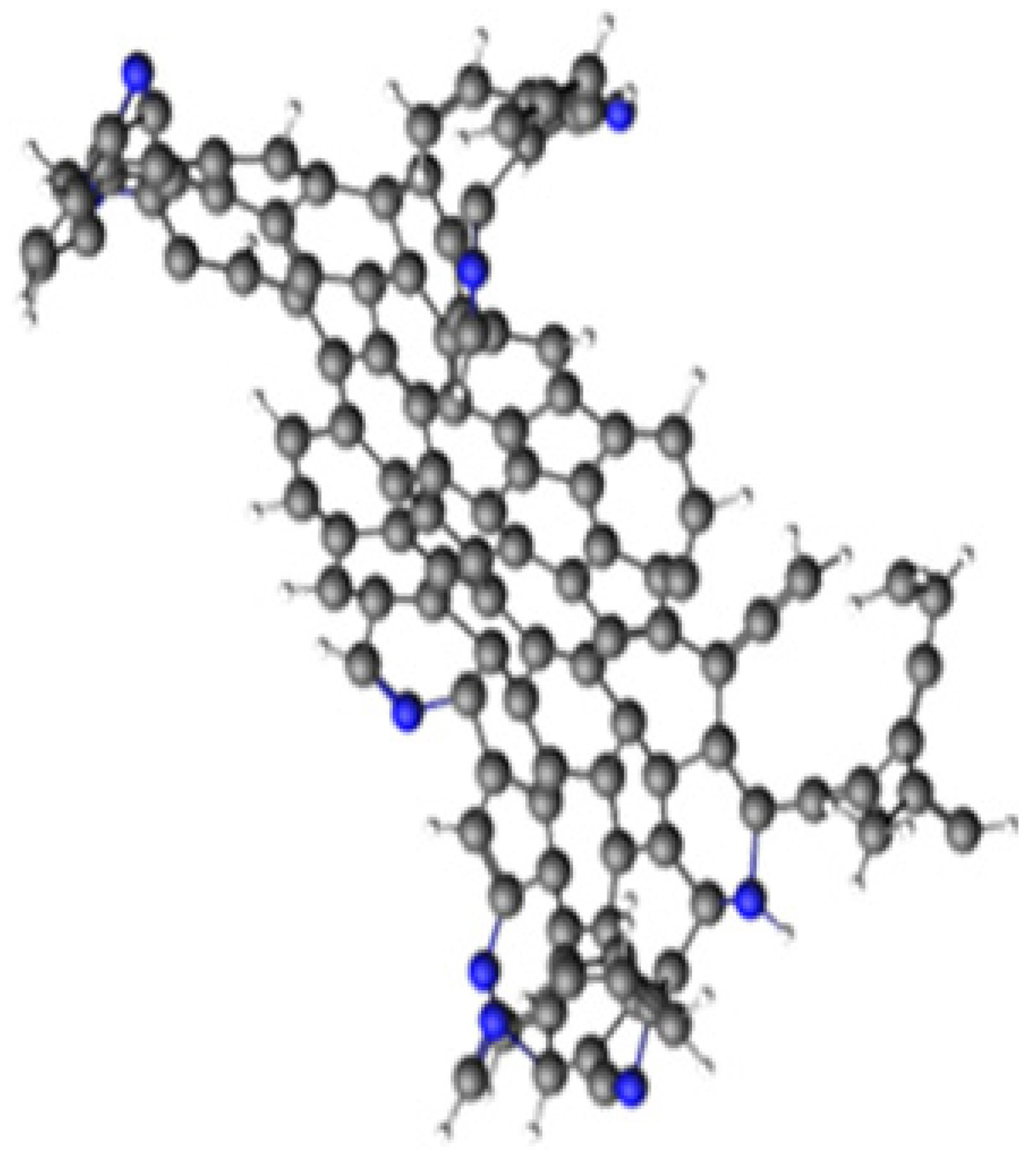	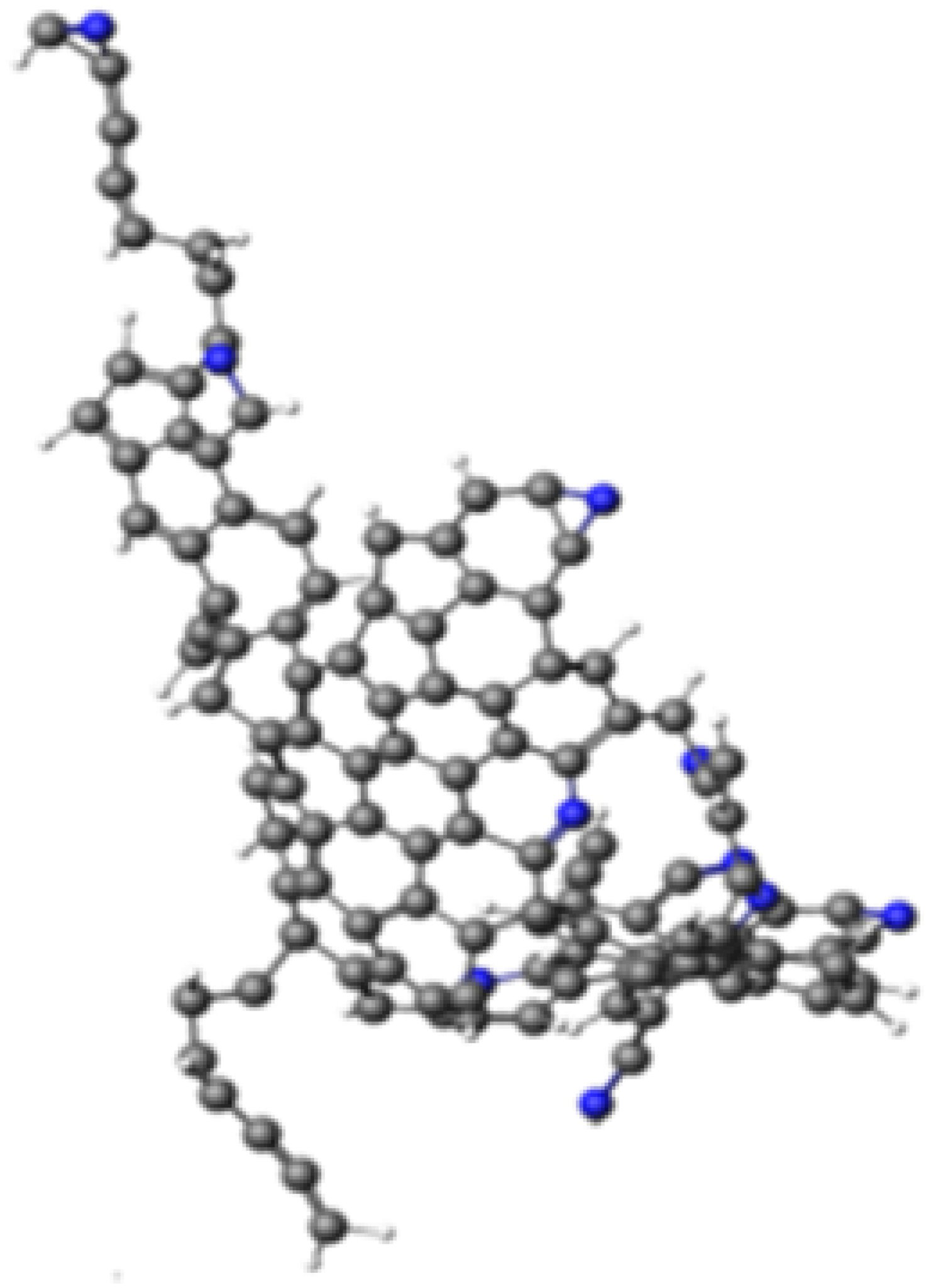
Molecular formula	C_133_H_42_N_8_	C_117_H_35_N_5_	C_119_H_34_N_6_	C_122_H_43_N_9_	C_124_H_39_N_10_
C/H atomic ratio	3.17	3.34	3.50	2.84	3.18
N/C atomic ratio	0.06	0.04	0.05	0.07	0.08
Number of benzene rings	21	21	21	17	26

**Table 3:** Simulation parameters for ReaxFF MD calculations.

Case	1-C_4_H_8_	2-C_4_H_8_	i-C_4_H_8_	n-C_4_H_10_	n-C_4_H_10_	NH_3_	O_2_	*T*, K	$\rho ,\;g/{\mathrm{cm}}^{3}$	φ
1	80	0	0	0	0	0	120	2800	0.3	4
2	80	0	0	0	0	40	130			
3	0	80	0	0	0	0	120			
4	0	80	0	0	0	40	130			
5	0	0	80	0	0	0	120			
6	0	0	80	0	0	40	130			
7	0	0	0	80	0	0	130			
8	0	0	0	80	0	40	140			
9	0	0	0	0	80	0	130			
10	0	0	0	0	80	40	140			

## Data Availability

The datasets presented in this article are not readily available because the data belong to confidential content as part of the research supported by the National Natural Science Foundation of China, and relevant data access is restricted in accordance with the project confidentiality management requirements. The data presented in this study are available on request from the corresponding author.
